# Constructing the national image of “China” in press conference interpreting: a corpus-based critical metaphor analysis

**DOI:** 10.3389/fpsyg.2026.1825833

**Published:** 2026-05-21

**Authors:** Congran Zhang

**Affiliations:** School of Foreign Languages, Renmin University of China, Beijing, China

**Keywords:** conceptual metaphor, corpus, critical metaphor analysis, interpreting strategies, press conference interpreting

## Abstract

**Introduction:**

This study investigates the role of conceptual metaphors in constructing China’s national image within Chinese-English press conference interpreting.

**Methods:**

Drawing on Critical Metaphor Analysis and the online corpus tool Wmatrix, the research identifies and examines metaphorical patterns in the interpreted diplomatic discourse.

**Results and discussion:**

Six types of conceptual metaphors relating to “China” emerge in the interpreting data, with the human and journey metaphors occurring most frequently, followed by building, ecology, war, and entertainment metaphors. These metaphors correspond to six cognitive frames whose linguistic realizations generally construct a favorable national image. The image schemata profile China as a traveler on a journey of struggle, a contributor to the international community, a builder of global causes, a guardian of the global environment, a fighter in challenging endeavors, and a performer on the world stage. Influenced by individual and social resources, three interpreting strategies are deployed, revealing an overall aim to render the national image more relatable, vivid, and warm while emphasizing China’s importance in the international community. These findings extend critical metaphor research on press conference interpreting by illuminating how metaphorical framing contributes to image construction. They also offer practical insights for interpreter training, demonstrating that strategic metaphor choices can enhance the communicative effectiveness of diplomatic discourse while maintaining fidelity to institutional positions.

## 1 Introduction

Nations are “an international system consisting of a group of interacting behavior units” (Boulding, 1959, p. 120) and are often understood as “imagined communities” (Anderson, 2006) constructed partly through cultural representations of belonging and discourses of national identity (Hogan, 2010, p. 63). In the contemporary global landscape, national image has become a crucial component of a country’s soft power (Liu et al., 2022, p. 2152). It refers to “the total cognitive, affective, and evaluative structure of the behavior unit, or its internal view of itself and its universe” (Boulding, 1959, p. 120). As a cognitive construct residing within the “black box” of the human mind, national image encompasses both a country’s self-perception and the perceptions held by other members of the international community (Dai and Hassan, 2023, p. 3191). Although it exists at the level of perception, national image can be observed through discursive constructions, particularly through identity narratives produced in political discourse (Wodak and Reisigl, 1999, p. 176), whose continuous reproduction shapes and reshapes a nation’s worldview (Martin, 1995).

As a structured form of information capital, national image is developed through the interaction of internal messages and external evaluations (Boulding, 1959, p. 121; Yang and Wang, 2023, p. 545). In China’s case, the annual press conferences of the Premier and the Foreign Minister serve as a key platform for communicating national policies, achievements, and future initiatives to the international community. Political discourse in such institutional contexts constitutes political acts, including governance, policy communication, and agenda-setting (Van Dijk, 2003, p. 212; Wodak et al., 2009, p. 80). Through these discursive practices, press conferences contribute to the construction of China’s national image.

As coordinators of cross-cultural communication, official interpreters play a crucial role in conveying China’s attitudes, stances, and viewpoints to international audiences. Despite the importance of perceptions in national image construction (Dai and Hassan, 2023, p. 3191), relatively few interpreting studies have examined the interaction between human cognition and the discursive construction of national images. Under frameworks such as Systemic Functional Linguistics (Halliday, 1985) and Critical Discourse Analysis (Fairclough, 1995), existing research either analyzes translated political discourse (Liu et al., 2022; Dai and Hassan, 2023; Yang and Wang, 2023) or investigates the cognitive processes of interpreting through empirical methods (Seeber and Kerzel, 2012). The relationship between cognitive mechanisms and discursive representation remains underexplored.

Conceptual Metaphor Theory (Lakoff and Johnson, 1980) provides an effective analytical lens for examining press conference discourse. Metaphor enables speakers to express intentions indirectly while shaping audience perceptions, making it particularly suited to the subtle and diplomatic nature of political language. Based on a self-compiled parallel corpus and the online semantic domain analysis tool Wmatrix, this study adopts Critical Metaphor Analysis to investigate conceptual metaphors in Chinese-English press conference interpreting. The aim is to reveal the role of conceptual metaphor interpretation in the construction of China’s national image.

## 2 Literature review

### 2.1 National image construction in institutional translation and interpreting

Recent years have witnessed a growing body of research that reconceptualizes institutional translators and interpreters as active ideological mediators rather than neutral conduits (Hu and Hye, 2020; Pan et al., 2020; Gu, 2022). Drawing on Critical Discourse Analysis (CDA) and corpus linguistics, these studies have examined how linguistic choices in translation and interpreting contribute to the reconstruction of political discourse, national narratives, and power relations. Pan et al. (2020) employed a corpus-based Critical Discourse Analysis framework to compare institutional translations with individual translations. The study identified three recurrent ideological shifts, namely shifts in interacting with readers, in representing actions, and in identifying participants (Pan et al., 2020, p. 51). These shifts make translations more reader-friendly and target-oriented while aligning with institutional agendas (Pan et al., 2020, p. 68). The study’s main contribution lies in providing empirical evidence of systematic ideological mediation in institutional translation, demonstrating how translation serves the political goal of “presenting China to the world” (Pan et al., 2020, p. 70). Gu (2022) investigates the interpreter-mediated premier-meets-the-press conferences through a corpus-based CDA approach. By focusing on the keyword cluster develop* and its collocational patterns, the study demonstrates how interpreters reinforce and recontextualize China’s “development” discourse within the broader meta-discourse of Reform and Opening-up (Gu, 2022, p. 65). The findings reveal that interpreters tend to show greater alignment by producing various lexical items related to key themes and concepts such as development, economy, and reform that constitute the broader RoU meta-discourse (Gu, 2022, p. 77). This study foregrounds interpreters’ discursive ownership and their role in shaping sociopolitical reality, which challenges the traditional view of interpreting as a neutral, mechanical process. It also highlights how interpreter-mediated discourse contributes to the global dissemination of China’s national image and may even influence geopolitical knowledge production.

Building on this empirical work, Gu (2023) adopted a mixed-methods approach integrating Critical Discourse Analysis (CDA) and corpus linguistics to propose a layered analytical framework for examining translators’ and interpreters’ ideological language use. The framework consists of multiple analytical “prongs” (e.g., lexical choices, established discourse categories, corpus-driven patterns, and close ST-TT comparison), combining top-down and bottom-up methods (Gu, 2023, p. 1027). Its main contribution lies in offering a comprehensive methodological model that addresses the lack of macro-level guidance in corpus-based CDA of bilingual discourse and enhances analytical rigor in translation and interpreting studies.

Further extending the scope to diachronic analysis, Pan et al. (2024) employed a corpus-based discourse analysis combining quantitative tools (e.g., AntConc, ParaConc) with Critical Discourse Analysis (CDA) to conduct a diachronic and contrastive study of CPC Work Reports and their English translations. The study found a clear diachronic shift in pronoun use, whereby translations increasingly construct a “closer and more symmetrical relationship with the target readers,” alongside a shift of “we + modal verbs” from expressing obligation to inclination (Pan et al., 2024, p. 924). These changes are interpreted as reflecting broader ideological evolution and audience reorientation (Pan et al., 2024, p. 924). The main contribution lies in demonstrating how micro-level linguistic features, such as pronouns, can index macro-level ideological change from a diachronic perspective.

These studies establish several key insights. First, institutional translators and interpreters are active agents of ideological mediation, whose linguistic choices contribute to the reconstruction of political discourse and national narratives. Second, ideological mediation operates across multiple linguistic levels, including keywords, collocations, pronouns, modality, and metadiscourse, which can be systematically investigated through the integration of CDA and corpus linguistics. Third, institutional contexts impose normative and ideological constraints that result in relatively consistent and aligned discourse production. Finally, recent methodological developments emphasize the importance of multi-layered, corpus-based, and diachronic approaches to capturing the complexity of ideological mediation in bilingual discourse.

While these studies have significantly advanced the investigation of institutional interpreters’ ideological mediation, relatively little attention has been paid to the specific role of conceptual metaphors in this process. Metaphors, as argued by Charteris-Black (2004, p. 88), “permit the hearer to infer meanings and have a subliminal role in facilitating hearers to find their own hopes, expectations, and beliefs,” and “have the potential for a socially influential and ideologically grounded role in political discourse.” The present study addresses this gap by applying Critical Metaphor Analysis to investigate how conceptual metaphors in press conference interpreting contribute to the ideological construction of China’s national image.

### 2.2 Conceptual metaphor and critical metaphor analysis

Conceptual Metaphor (CM) (Lakoff and Johnson, 1980) is a cognitive linguistic term proposed by George Lakoff and Mark Johnson. A conceptual metaphor maps the base scheme of a well-known tangible and bodily experienced source domain (D1) onto a more difficult-to-understand abstract target domain (D2) (Mannoni, 2021, p. 200).

As a paradigm of discourse analysis, Critical Discourse Analysis (CDA) began with the study of literary texts and was gradually applied to the analysis of non-literary texts, particularly political discourse. It highlights the relationship between discourse and society (Xia and Lin, 2020, p. 33). Critical Metaphor Analysis (CMA) has become a new approach to metaphor analysis that “aims to reveal the covert and possibly unconscious intentions of language users” (Charteris-Black, 2004, p. 34). CMA provides a way of unveiling the underlying ideologies, attitudes, and beliefs of metaphorical expressions. It constitutes a vital means of understanding the complex relationships between language, thought, and social context (Charteris-Black, 2004, p. 42). Moreover, in political discourse, CMA views metaphors as a crucial means of conceptualizing political issues and constructing worldviews (Charteris-Black, 2004, p. 48).

**Figure 1:**
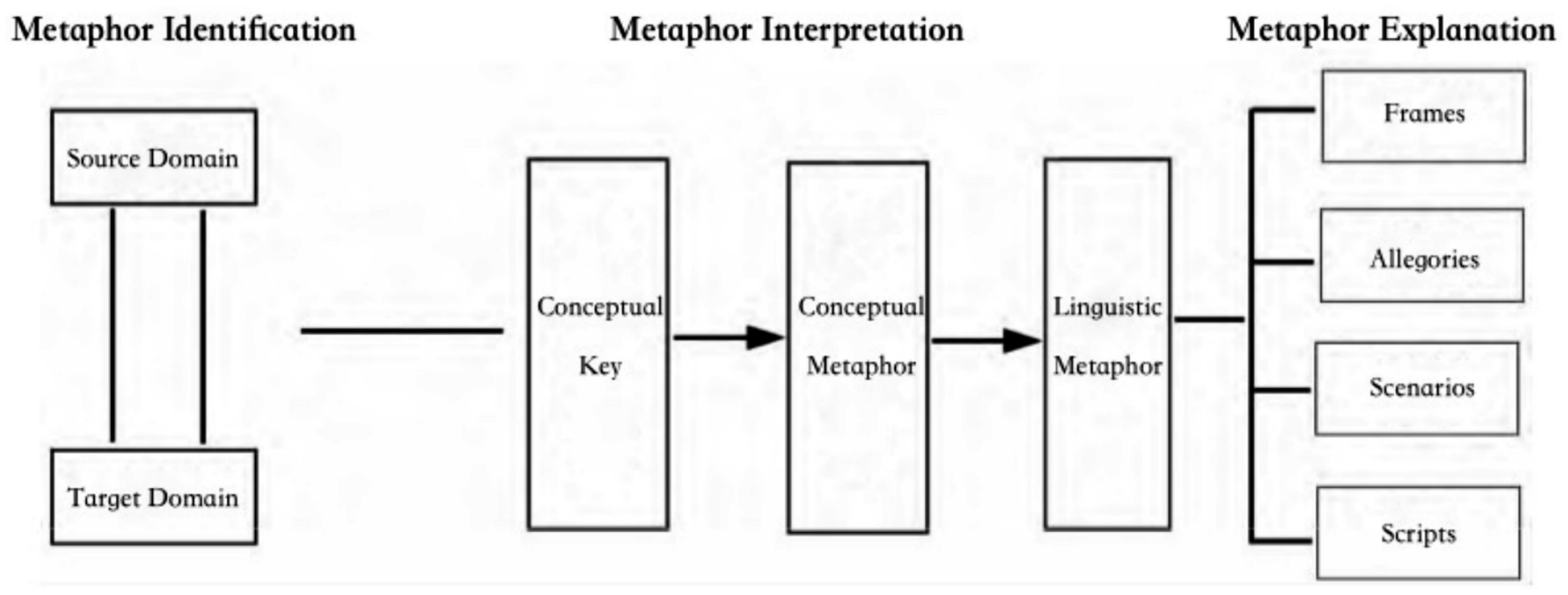
Critical metaphor analysis model.

Based on Halliday’s (1985) functional linguistics and derived from Fairclough’s (1995) methodology of CDA, CMA contains three stages (Cameron and Low, 1999, p. 88): collecting examples of linguistic metaphors (metaphor identification), generalizing from them to the conceptual metaphors they exemplify (metaphor interpretation), and using the results to suggest understandings or thought patterns that construct or constrain people’s beliefs and actions (metaphor explanation). Sun and Xiong (2022) have developed a theoretical model for CMA, illustrated in Figure 1. They introduced the “conceptual key” to metaphor interpretation and four linguistic concepts to elucidate the process of metaphor explanation (Sun and Xiong, 2022, p. 4). To explain metaphors, their model relies primarily on the “framing” theory. Entman (1993) has comprehensively defined framing:

“Framing essentially involves selection and salience. To frame is to select some aspects of a perceived reality and make them more salient in a communicating text, in such a way as to promote a particular problem definition, causal interpretation, moral evaluation, and/or treatment recommendation for the item described (Entman, 1993, p. 52).”

Despite the profound research background of CMA, relevant studies are scarce in conference interpreting. Most existing critical metaphor studies focus on political discourse, for example, the speeches of national leaders and the news coverage of political events (Wu and Li, 2023, p. 92). Charteris-Black (2004) comprehensively conducted a CMA of the political discourses in the New Labor manifestos, British party-political manifestos, and American presidential speeches. He classified metaphors in political discourse into eight types: conflict metaphors, building metaphors, journey metaphors, plant metaphors, religious metaphors, light and fire metaphors, physical environment metaphors, and body part metaphors (Charteris-Black, 2004, p. 45–109). Moreover, there are few translation studies on the CMA of political discourse. Most scholars have focused on the schema structure of conceptual metaphors within one language, most commonly English (Wu and Pang, 2011; Wu et al., 2020; Sun and Xiong, 2022). Insufficient attention has been paid to the disparities in semantic domains during language translation and cross-cultural processes. The ideologies embedded within the code-switching processes are worth discussing.

In addressing this gap, Li and Li (2015) examined “metaphorisation” as one type of shift in Chinese political translation through a diachronic, corpus-informed textual analysis of 15 political speeches and their English translations from the 1970s to the 2010s. By manually identifying target-oriented translation shifts, they showed that metaphorisation involves rendering neutral source expressions into more vivid metaphorical forms. This study contributes by demonstrating that metaphor use in political translation “functions as a medium to connect individual understanding with the immediate socio-cultural experience of the metaphor producer (Zinken, 2003),” and “conjures up a concrete image which may intensify the positive reception of the rendering” (Li and Li, 2015, p. 430). Their findings indicate that such shifts are particularly frequent in the Deng Xiaoping period, where translators employed metaphors to enhance rhetorical appeal and international communicative effectiveness (Li and Li, 2015, p. 434).

Complementing this line of inquiry, Song and Zhang (2023) conducted a critical metaphor analysis of metaphor shifts in the English translation of the Communist Party of China’s centenary speech, drawing on van Dijk’s (1998) Ideological Square Model (ISM). Their study identified six types of metaphors (journey, war, building, body, cause, and illness) in both the source and target texts, finding that while the overall number of metaphors was largely preserved, systematic shifts occurred through substitution (replacement or modification) and cancelation (Song and Zhang, 2023, p. 2). These metaphor shifts are not merely linguistic or cultural adjustments but are ideologically mediated by the self-serving principle inherent to institutional translation (Song and Zhang, 2023, p. 12). More importantly, they proposed incorporating the traditional Chinese philosophy of *He* (harmony) into the ISM framework to better account for the strategic mitigation of confrontational metaphors in the English version, which serves to present a firm yet non-aggressive national image to an international audience (Song and Zhang, 2023, pp. 11–13). Their work thus advances CMA by extending its application from political discourse analysis to translation studies, and by demonstrating how ideological mediation operates in cross-cultural adaptation.

### 2.3 Corpus-based metaphor analysis

The sole evidence that Lakoff and Johnson (1980) presented to support the conceptual metaphor theory was their “introspections” about carefully selected examples (Shutova et al., 2013, p. 1264). Such introspective evidence in no way guarantees that the epistemological account is accurate, and in some cases, it may be downright misleading (McGlone, 2007, p. 115). The application of corpus to metaphor analysis has been proposed by Charteris-Black (2004). As he noted, corpora can combine quantitative and qualitative approaches to investigating metaphors: Qualitative judgments can establish what will be counted as a metaphor, and quantitative analysis allows us to measure the frequency of a metaphor in a corpus and to estimate the extent to which a particular metaphorical sense of a word form has become conventionalized (Charteris-Black, 2004, p. 34).

Since then, a series of critical metaphor studies have been carried out using corpus tools. They either concentrate on the metaphors associated with specific words (Bas, 2020) or delve into metaphorical expressions related to a particular topic (Wu and Pang, 2011; Wang and Xin, 2019; Liu and Cai, 2023). The “word frequency,” “collocation,” and more data retrieved from a corpus can help to discern metaphorical expressions and explain their semantic meanings.

Existing corpus research employs two approaches: “corpus-driven,” exploring all metaphors without predefined conceptual metaphors, and “corpus-based,” searching for words or expressions relevant to the predefined conceptual metaphors. The former may lack generalizability due to limited corpus size, while the latter risks overlooking unsearched metaphors and lacks non-linguistic contextual information (Sun, 2013, p. 11). Most existing studies use the latter approach, investigating certain words or expressions. As a conceptual metaphor is the mapping across different cognitive domains, the corpus-based methods for identifying conceptual metaphors are mainly used to retrieve source and target domain words. For example, after analyzing a small corpus, Koller (2004) concluded the conceptual metaphor “business is war” through a retrieval approach of the words collocated with “war.” This is a corpus retrieval method based on source domain words.

With technological development, researchers have achieved automatic machine annotation of metaphors. Koller et al. (2008) conducted a computer-aided metaphor analysis of discourse in different domains. They realized automatic coding of semantic domains through the UCREL Semantic Annotation System (USAS) embedded in the corpus analysis tool Wmatrix. USAS can automatically assign semantic domain tags to texts (Rayson, 2008, p. 527), enabling researchers to directly search and extract metaphorical expressions in the database and avoid the omission of manual recognition of candidate metaphors as much as possible (Wu and Li, 2023, p. 91). Wmatrix-based metaphor identification is also focused on the source and target domains. The identification process involves three key steps: First, generating a semantic domain table; Second, retrieving each type within the top semantic domains and its concordance; Third, using the Metaphor Identification Procedure (MIP) proposed by Pragglejaz Group (2007) to identify metaphors associated with each type (Sun, 2013, p. 13–15).

### 2.4 Metaphorical cognitive mechanisms

According to Charteris-Black (2004), from a cognitive semantic perspective, image schemas are the source of metaphorical mappings for abstract domains derived from everyday bodily interaction with the physical environment. In short, with basic-level domains, image schemas constitute the material for conceptualization (Charteris-Black, 2004, p. 14). According to the “Invariance Principle” (Lakoff, 1993), schema structure is the cognitive topology of the source domain preserved by metaphorical mappings, which is consistent with the inherent structure of the target domain (Lakoff, 1993, p. 215). The cognitive perspective of this study is centered on exploring the schema structures underpinning metaphorical expressions.

According to Tan (2016), a conceptual metaphor contains an internally coherent system with its derived linguistic metaphor (metaphorical expression). Such systematicity is related to interpreting D1 and D2, the two poles of the conceptual mapping. With the schema-instance relationship at the core and the topological structure, they can form a network system around the mapping schema (Tan, 2016, p. 97). Metaphorical expressions can activate the schema structure in people’s minds. In the interpretation process, things with salient status in the scene are often selected as activators to the schema and thus encoded as the main constituent symbols of metaphorical expression (Tan, 2016, p. 98). In other words, linguistic forms prompt cognitive processes. Then, Tan (2023) proposed the base-profile relationship within the cognitive domains. He noted that people’s cognition of the relationship between source texts and target texts is facing a shift from “schema-instance” to “base-profile” (Tan, 2023, p. 754–755). Based on Langacker’s theory (1987), the concept explicitly designated by a linguistic expression corresponds to the “profile” in the “base.” “Base” is the underlying matrix of the cognitive domain that needs to be activated to understand the expression. “Profile” is the highlighted substructure within the base, attached to the base by the process of “profiling” (Tan, 2023, p. 752). Different expressions can activate the same cognitive domain, which reflects the various profiling angles within one cognitive base. Different profiling methods can also result in subtle differences in the semantic meanings.

**Figure 2:**
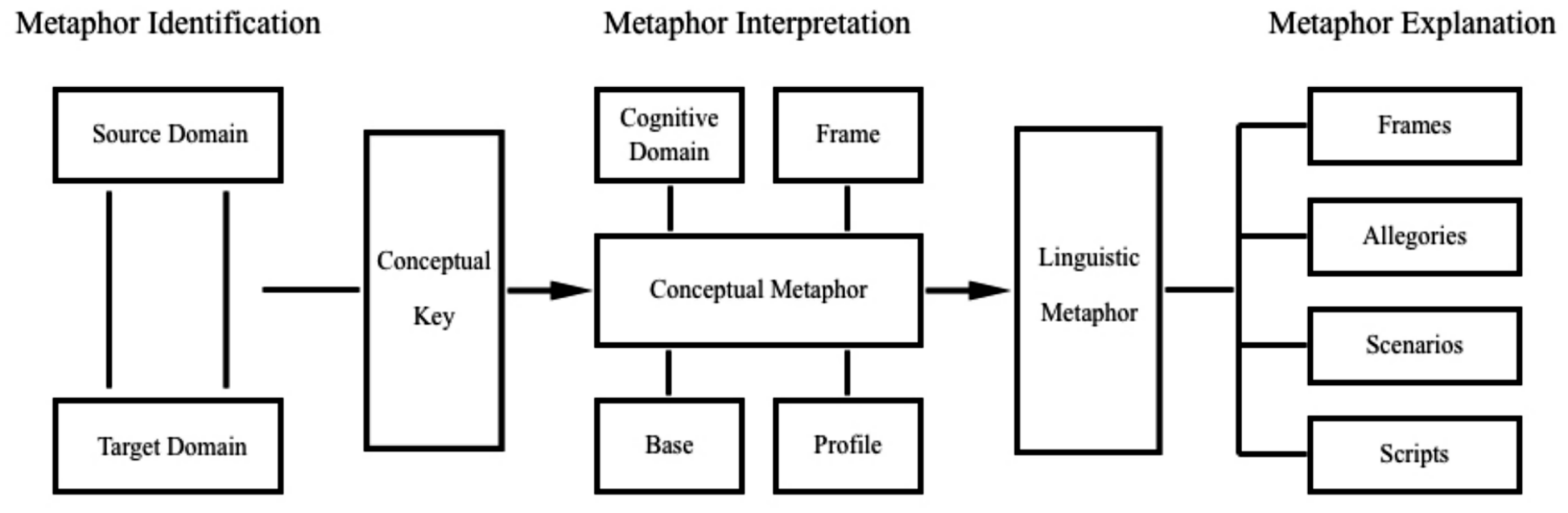
Adapted CMA model.

Linguistic expressions are the index to activate the underlying cognitive frame. Within the frame, different profiles can correspond to one base of the cognitive domain. “Expression” and “scene” are the onstage symbols, while “frame,” “profile,” “base,” and “cognitive domain” constitute the backstage cognition. This study revises the metaphor interpretation section of the previous CMA model (Sun and Xiong, 2022) and proposes an adapted theoretical model for CMA, illustrated in Figure 2. Based on this model, this study will explore questions including which frame the interpreter has activated, the profiling techniques they have employed, and the underlying intentions reflected by their chosen cognitive domain.

## 3 Research design

### 3.1 Research questions

According to the general research objectives and the adapted CMA model, three research questions are raised, covering “Metaphor Identification,” “Metaphor Interpretation and Explanation,” and “Interpreting Strategies”:

*RQ1* (Metaphor Identification): What are the conceptual metaphors related to “China” in the interpreting discourse from 2014 to 2023?

*RQ2* (Metaphor Interpretation and Explanation): What cognitive mechanisms and ideologies underlie these conceptual metaphors, and what kinds of national images of China are constructed by these metaphors in the interpreting discourse?

*RQ3* (Interpreting Strategies): What interpreting strategies are deployed for different metaphors, and what factors influence the metaphor choices in the interpreting discourse?

### 3.2 Research data

The corpus under research is the Chinese-English Press Conference Interpreting Corpus. This bilingual parallel corpus collects transcripts of the Chinese-English consecutive interpreting in the press conferences held after the National People’s Congress (NPC) from 2014 to 2023. These press conferences were presided over by the Chinese Premier and Foreign Minister, including Premier Li Keqiang’s press conferences from 2015 to 2022, Foreign Minister Wang Yi’s press conferences from 2014 to 2022, and Premier Li Qiang’s press conference in 2023. A total of 18 press conferences are included, with detailed corpus capacity information presented in Table 1.

**Table 1:** Capacity of Chinese-English Press Conference Interpreting Corpus.

Sub-corpora	Types	Tokens	TTR	STTR
Chinese-simplified	9,918	87,627	11.32	78.06
English	8,680	120,709	7.19	71.47

The period selection for the corpus under research, from 2014 to 2023, is primarily based on the following three considerations. First, a decade-long period provides sufficient time to capture and observe trends and patterns of metaphorical expressions, without being overly extensive and unmanageable. Second, the years 2014–2023 mark a dynamic period in China’s international status and influence, with significant events such as the advancement of the Belt and Road Initiative, changes in China-US relations, and the impact of the COVID-19 pandemic. These events may have significantly influenced the metaphorical expressions related to China in international discourse as China navigated the turbulent circumstances of unprecedented global changes unseen in a century. Last, the data collected spans from the 12th National People’s Congress to the opening of the 14th National People’s Congress in 2023, ensuring the timeliness of this study, which provides insights into current and future international relations and foreign policies. By examining metaphorical expressions during this period, this study aims to uncover the general trends of the latest discursive construction of China’s national image in the interpreting context.

Moreover, one important assumption for the data collection process is that the institutional interpreting norms were consistent across all 18 press conferences. Since the interpreters for these 18 press conferences were officially appointed by the Chinese government, the interpreting norms, level of formality, stylistic register, and political stance are assumed to be consistent. It is also assumed that there has been minimal deviation in employing metaphorical expressions due to personal habits or stylistic differences.

This study first employs the semantic tagging feature of Wmatrix. The semantic tagset includes 21 semantic domains, shown in Table 2. These domains can be further subdivided into 232 subdomains. For example, the domain tag “I” (money and commerce in the industry) can be subdivided into “I1” (money generally), “I2” (business), “I3” (work and employment), and “I4” (Industry).

**Table 2:** USAS semantic tagset.

Tags	Semantic domains
A	General and Abstract Terms
B	Human Body
C	Arts and Crafts
E	Emotions
F	Food and Agriculture
G	Government and Politics
H	Architecture, Houses and Buildings
I	Industry and Money
K	Entertainment and Sports
L	Life and Living Things
M	Moving and Location
N	Numbers and Measurement
O	Substances, Materials and Objects
P	Education
Q	Language and Communication
S	Social Actions
T	Time
W	World and Environment
X	Psychological Actions, States and Processes
Y	Science and Technology
Z	Names and Grammar

The reference corpus used is BNC Sampler Spoken embedded in Wmatrix. The interpreting corpus is compared with the reference corpus by setting the log-likelihood ratio cut-off at 6.63. The result is a table of the top 20 thematic semantic domains sorted in descending order by the LL values, as presented in Table 3.

**Table 3:** Top 20 thematic semantic domains in press conference interpreting.

Number	Item	O1	%1	O2	%2	LL	LogRatio	Semtag
1	Z2	3,621	3.16	3,541	0.36+	7205.82	3.13	Geographical names
2	Z99	3,144	2.74	5,684	0.58+	3956.04	2.24	Unmatched
3	S8+	1986	1.73	2020	0.21+	3861.20	3.07	Helping
4	G1.1	1,524	1.33	1,084	0.11+	3580.51	3.59	Government
5	M7	2038	1.78	3,261	0.33+	2862.23	2.42	Places
6	A2.1+	1,152	1.00	2031	0.21+	1484.10	2.28	Change
7	A11.1+	787	0.69	955	0.10+	1366.12	2.82	Important
8	W3	654	0.57	699	0.07+	1233.52	3.00	Geographical terms
9	Z5	35,076	30.56	252,254	25.67+	897.00	0.25	Grammatical bin
10	W1	386	0.34	300	0.03+	869.14	3.46	The universe
11	I2.1	640	0.56	1,086	0.11+	853.77	2.34	Business: Generally
12	A2.2	809	0.70	1891	0.19+	773.97	1.87	Cause & Effect/Connection
13	S1.1.2+	415	0.36	471	0.05+	753.35	2.92	Reciprocal
14	N3.2+	538	0.47	1,046	0.11+	630.46	2.14	Size: Big
15	X5.2+	416	0.36	612	0.06+	626.21	2.54	Interested/excited/energetic
16	A2.1-	201	0.18	72	0.01+	608.56	4.58	No change
17	I1.3-	280	0.24	237	0.02+	603.62	3.34	Cheap
18	S3.1	431	0.38	752	0.08+	560.61	2.29	Personal relationship: General
19	S1.2.5+	212	0.18	119	0.01+	551.24	3.93	Tough/strong
20	E3+	251	0.22	207	0.02+	548.48	3.38	Calm

**Figure 3:**
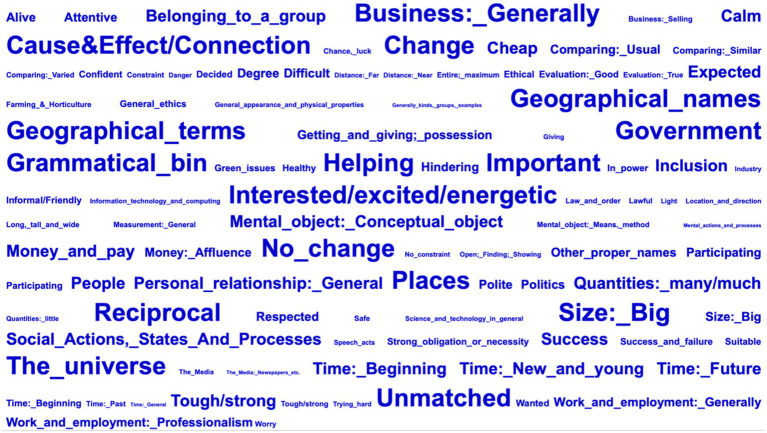
Word cloud of thematic semantic domains in press conference interpreting.

“O1” and “%1” represent the frequency and relative frequency of the semantic domain in the research corpus. “O2” and “%2” denote the frequency and relative frequency of the same semantic domain in the reference corpus. “+” indicates a significantly higher frequency in the research corpus compared to the reference corpus. “LL” represents the log-likelihood ratio value for the thematic semantic domain, indicating the degree of significance for the overuse of that semantic domain. In total, 243 thematic semantic domains are determined, as shown in Figure 3.

### 3.3 Analytical tools

This study employs a corpus-based method that combines qualitative and quantitative approaches. Following the analytical framework, this section outlines the research methods used at each analytical stage. In general, “Metaphor Identification” draws from Pragglejaz Group’s (2007) Metaphor Identification Procedure (MIP), facilitated by the online corpus analysis tool Wmatrix.^1^ “Metaphor Interpretation” is grounded in Langacker’s (1987) and Tan’s (2023) cognitive linguistic theories, especially the Profiling Theory. “Metaphor Explanation” is based on Entman’s (1993) Framing Theory and Sun and Xiong’s (2022) analytical theory for metaphor explanation. Wmatrix is also employed to discern the semantic domains of specific expressions. The discussion on “Interpreting Strategies” is inspired by Sheng’s (2021) categorization of metaphor interpreting strategies. This study also employs the parallel corpus annotation tools in Visual Studio Code to annotate different interpreting strategies.

The metaphor identification of this study is assisted by the online corpus analysis tool Wmatrix. After processing the corpus with Wmatrix, each expression in the interpreting discourse is categorized into a semantic domain, with the source domain words being potential metaphorical expressions. While tools like WordSmith identify metaphors based on words, Wmatrix-based identification is grounded in semantic domains, which better reflects the nature of conceptual metaphor as a mapping between different cognitive domains (Sun, 2013, p. 12). The metaphor identification process start from the target domain (i.e., the Z2 domain where the term “China” is located) to see how the interpreting discourse employs conceptual metaphors to construct China’s national image. The top ten types in the “Z2” domain are shown in Table 4. The largest semantic domain in the corpus is Z2 (Geographical Names), within which the term “China” appears 1,429 times, significantly higher than any other term, with numerous conceptual metaphors found in its contextual collocations. As this term bears a direct relation to China’s national image, examining its related conceptual metaphors and interpreting strategies is crucial for effectively constructing China’s national image.

**Table 4:** Top 10 types in Z2 domain.

Word	Semtag	Frequency	Relative frequency
China	Z2	1,429	1.25
Chinese	Z2	432	0.38
China’s	Z2	243	0.21
Taiwan	Z2	124	0.11
Hong_Kong	Z2	109	0.09
United_States	Z2	98	0.09
Africa	Z2	81	0.07
Russia	Z2	67	0.06
Japan	Z2	65	0.06
South_China	Z2	60	0.05

The Wmatrix-assisted Metaphor Identification process can be divided into three steps: setting the target domain; applying the Metaphor Identification Procedure (MIP) (Pragglejaz Group, 2007); and annotating the source domain (Sun, 2013, pp. 13–14). The first step of setting the target domain (Z2, “China”) has already been completed, with the next step being the application of MIP. The procedure involves manually clicking on the concordance lines for the word “China” and reading them line by line. A selected part of these concordance lines is shown in Table 5.

**Table 5:** Part of concordance lines for “China.”

re world and benefit all humanity. **China** is a firm believer in making COVID v	1,116
king COVID vaccines a public good. **China** was among the first to pledge that i	1,117
be made a global public good, and **China** has worked in real earnest to improv	1,118
rdability in developing countries. **China** is a committed front-runner in promo	1,119
ning recognition across the world. **China** is prepared to discuss with other co	1,120
mutual recognition of vaccination. **China** is a steadfast advocate for equitabl	1,121
We have joined COVAX, under which **China** has undertaken to provide an initial	1,122
in developing countries. So far, **China** has donated or is donating COVID vac	1,123
long as it is safe and effective. **China** opposes “vaccine nationalism”. We	1,124
t Xi Jinping to express support for **China**'s COVID response. The United Arab	1,125
s set a new example. Trade between **China** and Arab states has reached US$240 b	1,126
states has reached US$240 billion. **China** remains the largest trading partner	1,127
as roots and goodwill as branches. **China** will work with Arab states in solida	1,128

A methodological limitation of this study should be acknowledged. The metaphor identification process begins from the target domain of “China” (the Z2 semantic domain in Wmatrix) and examines the concordance lines for the node word “China” to identify metaphorical expressions that directly collocate with this term. This approach, while systematic and replicable, inevitably excludes metaphorical constructions of the nation that do not explicitly mention “China,” such as those mediated through pronouns (e.g., “we,” “our”), metonymic references (e.g., “Beijing,” “the Chinese government”), or other referential strategies. As previous research has shown (e.g., Gu, 2023; Song and Zhang, 2023; Pan et al., 2024), pronouns and metonyms are themselves significant sites of ideological mediation in political discourse. Future research could extend the analysis by examining how these other referential strategies co-occur with metaphorical expressions or function independently as vehicles for metaphorical framing.

Notwithstanding this limitation, focusing on the node word “China” represents a defensible methodological choice. First, this approach offers a high degree of operational transparency. By anchoring the identification procedure in a clearly defined lexical item, the study allows other researchers to replicate the process with minimal ambiguity. This replicability is essential for corpus-based critical metaphor analysis, where consistency in identification across different analysts or datasets directly affects the reliability of findings. Second, the node-word method is scalable and adaptable. The same analytical workflow (e.g., selecting a target term, retrieving its concordance lines, and annotating metaphorical uses) can be applied to other discourse contexts, language pairs, or political entities without requiring substantial modifications to the core procedure. This enhances the methodological utility of the study for comparative or longitudinal research. Third, given the practical constraints of manual metaphor annotation, a fully exhaustive identification of all possible metaphorical references to a nation (including those mediated by pronouns, metonyms, or ellipsis) would require a more complex annotation scheme. Within the manageable scope of a single study, focusing on the most frequent and direct referential form strikes a practical balance between analytical depth and resource feasibility. The high frequency of “China” in the corpus (1,429 occurrences) ensures that this focused analysis still captures a substantial portion of the metaphorical framing of the nation. While the limitations acknowledged here warrant caution in generalizing the findings, they do not invalidate the contributions made within the defined scope of the investigation.

The main steps for metaphor identification using MIP are as follows (Pragglejaz Group, 2007, p. 3):

“1. Read the entire text discourse to establish a general understanding of the meaning.

2. Determine the lexical units in the text discourse.

3. (a) For each lexical unit in the text, establish its meaning in context, that is, how it applies to an entity, relation, or attribute in the situation evoked by the text (contextual meaning). Consider what comes before and after the lexical unit.

(b) For each lexical unit, determine if it has a more basic contemporary meaning in other contexts than the one in the given context. For our purposes, basic meanings tend to be

--More concrete [what they evoke is easier to imagine, see, hear, feel, smell, and taste];

--Related to bodily action;

--More precise (as opposed to vague);

--Historically older;

Basic meanings are not necessarily the most frequent meanings of the lexical unit.

(c) If the lexical unit has a more basic current-contemporary meaning in another context than the given context, decide whether the contextual meaning contrasts with the basic meaning but can be understood in comparison.

4. If yes, mark the lexical unit as metaphorical.”

For an example of Line 1,116 in Table 5, the text discourse “China is a firm believer” contains five lexical units: “China,” “is,” “a,” “firm,” and “believer.” The contextual meanings are, respectively: “China” refers to a country; “is” indicates existence; “a” denotes quantity; “firm” describes a mental state; and “believer” refers to a type of national image. Among them, “firm” and “believer” convey additional contextual meanings beyond their basic contemporary meanings. “Firm” originally refers to the quality of an object, instead of a mental state. “Believer” typically denotes a type of person, rather than a specific national image. The contextual meanings of these terms contrast with their basic meanings, which suggests their metaphorical usage (Sun, 2013, p. 14).

**Figure 4:**
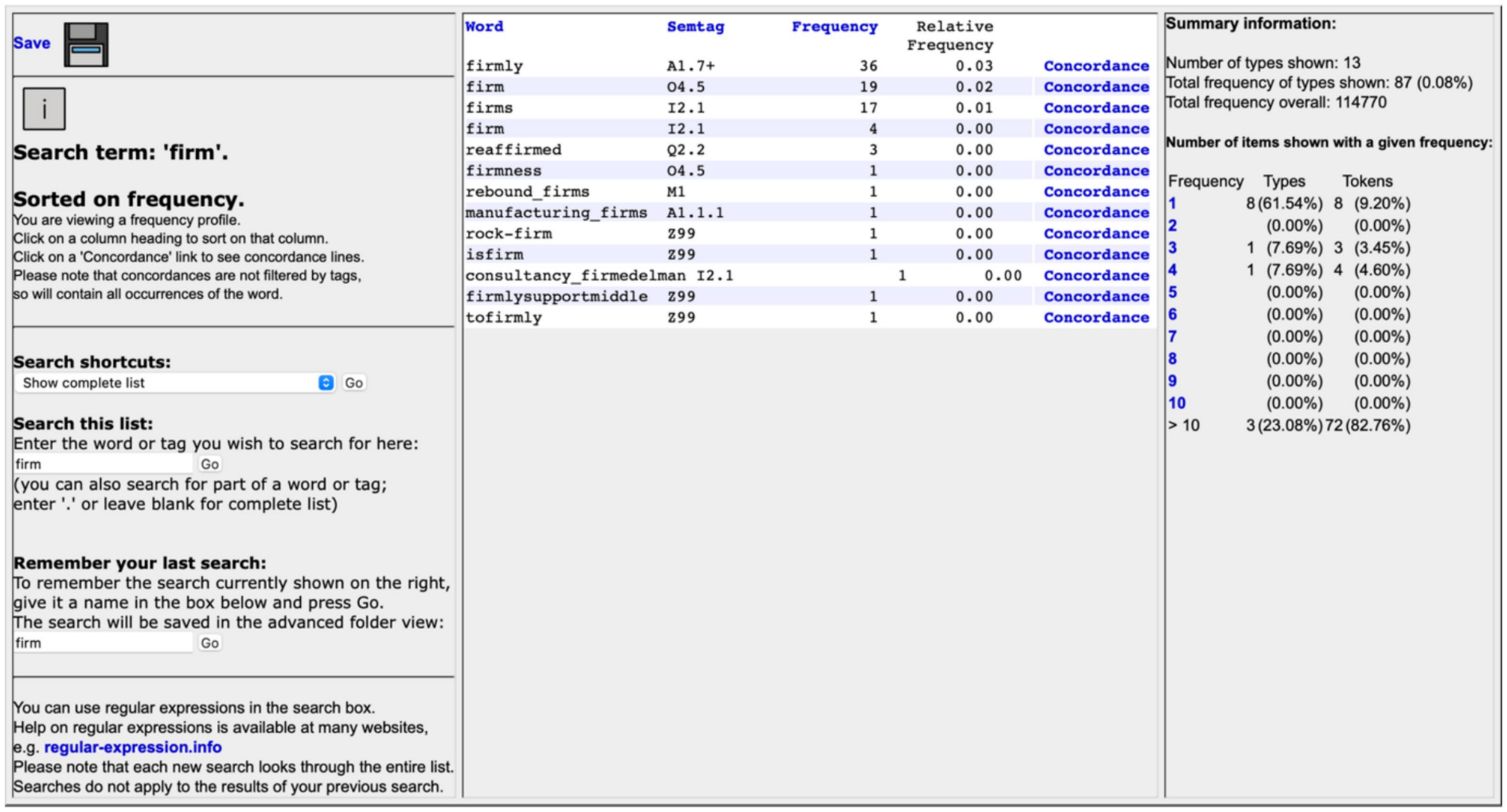
Retrieval of “Firm” in Wmatrix.

**Figure 5:**
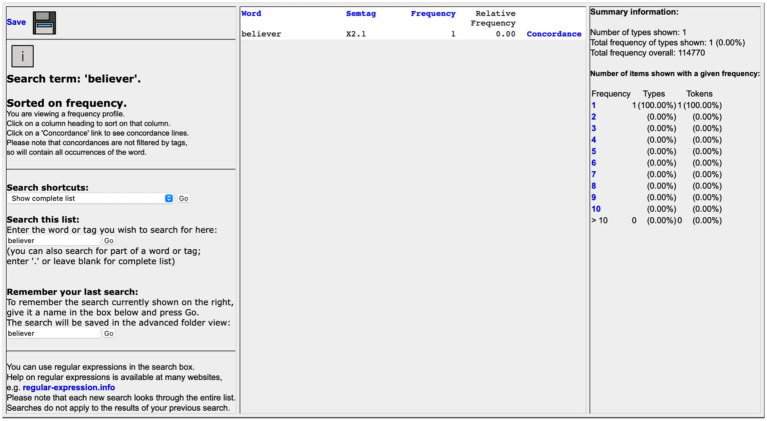
Retrieval of “Believer” in Wmatrix.

Based on the Metaphor Identification Process, this study integrates Wmatrix into the metaphor identification process. This involves supplementing manual reading of concordance lines with the retrieval of the semantic domains of specific lexical units in Wmatrix. The USAS semantic tagset embedded in Wmatrix indicates semantic domains for reference. For example, the semantic tags for “firm” and “believer” are shown in Figures 4, 5. The semantic tagset in Wmatrix annotates “firm” as “O4.5 (Texture)” and “believer” as “X2.1 (Thought, belief).” These two semantic domains signify two conceptual mappings. The O semantic domain (including substances, materials, and objects) indicates an ontological metaphor with the conceptual mapping from objects to mental qualities. The X semantic domain (including human psychological actions, states, and processes) projects a conceptual mapping to the Z2 (Geographical names) domain, with the conceptual metaphor CHINA IS A HUMAN. Therefore, “firm” and “believer” are identified as metaphorical expressions.

**Figure 6:**
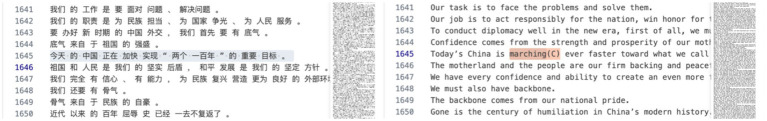
An example of corpus annotation in Visual Studio Code.

After all the conceptual metaphors in the English text are identified, interpreted, and explained, the next step is to investigate the interpreting strategies for conceptual metaphors in the bilingual parallel texts. These strategies are categorized into three types: Metaphor Creation (C), Retention (R), and Transformation (T). The corpus analysis tool Visual Studio Code is employed to annotate these strategies in the interpreting corpus. The corpus annotation process is manually conducted. An example is shown in Figure 6. After the corpus annotation, all metaphor-bearer words and their handling strategies are collected in a Microsoft Excel form for further discussion. For example, if the metaphor-bearer word “root” appears 27 times, including one Metaphor Creation, 20 Retentions, and six Transformations, in the form it appears as “root (27, C1, R20, T6).”

## 4 Results and discussion

### 4.1 Results of metaphor identification in press conference interpreting

The source domains, metaphor-bearer words, and their frequencies are summarized in Table 6. Due to space limits, Table 6 only lists part of the metaphor-bearer words. The M (Moving and Location) and S (Social Actions) domains are the largest source domains with the most metaphor-bearer words, with frequencies of 251 and 159, respectively.

**Table 6:** Source domains, metaphor-bearer words, and frequencies.

Source domains	Metaphor-bearer words	Frequencies
A (General and Abstract Terms)	unveil, inherit	4
B (Human Body)	arms, shoulder	29
C (Arts and Crafts)	paint, chord	3
E (Emotion)	calm, confidence	16
F (Food and Agriculture)	fruits/fruitful	6
G (Government and Politics)	battle, fortress	7
H (Architecture, Houses and Buildings)	neighbor, build/building	81
I (Industry and Money)	work, afford	55
K (Entertainment and Sports)	player, kick off	45
L (Life and Living Things)	wings, sapling	8
M (Moving and Location)	path, (all) along/alongside	251
N (Numbers and Measurement)	height, deepen	26
O (Substances, Materials and Objects)	banner, firmness	14
Q (Language and Communication)	response, persuade	50
S (Social Actions)	foster, host	159
T (Time)	milestone, rebirth	21
W (World and Environment)	breeze, rain	4
X (Psychological Actions, States and Processes)	determined/determination	47
Sum		826

Drawing on the Profiling Theory (Tan, 2023), this section categorizes metaphor-bearer words to classify the conceptual metaphors into six major types: human metaphors (324 occurrences), journey metaphors (274 occurrences), building metaphors (45 occurrences), ecology metaphors (12 occurrences), war metaphors (7 occurrences), and entertainment metaphors (45 occurrences). “Journey” and “human” are the two largest source domains of conceptual metaphors.

### 4.2 Metaphor interpretation and explanation

“Metaphor Interpretation” is employed to categorize metaphors and uncover the cognitive mechanisms underlying metaphorical expressions, including analyzing the cognitive frames, base-profile relationships, source domains, and target domains in the conceptual mappings. In this section, the metaphor interpretation starts from the M (Moving and Location) and S (Social Actions) domains, the two largest source domains. A key point is that the annotated semantic domain of a linguistic term is not necessarily the most inclusive source domain involved in the conceptual mapping. For example, “milestone” belongs to the T1.2 (Time: Momentary) domain, but the expression “This is a *milestone* in China-Russia relations” contains the conceptual metaphor THE DEVELOPMENT OF CHINA-RUSSIA RELATIONS IS A JOURNEY, of which the source domain “journey” is in the M1 (Moving, Coming, and Going) domain. From a cognitive linguistic perspective, the “cognitive base” (Langacker, 1987; Tan, 2023) of this metaphor can be subdivided into the “temporal” and “spatial” profiles of a journey. Therefore, higher-level cognitive domains must be identified from the semantic domains annotated by the USAS tagset to ensure the inclusiveness of conceptual metaphors and the diversity of metaphorical expressions.

“Metaphor Explanation” explores the relationship between conceptual metaphors and their social contexts, revealing the underlying social ideologies and power dynamics embedded within them (Sun and Xiong, 2022, p. 3). A critical analysis of metaphor necessitates moving beyond mere identification to interrogating how conceptual metaphors function as instruments of ideological reinforcement and the negotiation of power within specific social contexts. From a critical perspective, metaphors are not simply rhetorical flourishes but are powerful tools with strong “framing” capabilities, actively constructing “frames” (Entman, 1993), “scenarios” (Musolff, 2016), “scripts” (Schank and Abelson, 1977), and “allegories” (Sun and Xiong, 2022, pp. 3–4) that delimit permissible interpretations. “Scenarios” function as “figurative mini-narratives” that do more than represent reality; they impose a particular “evaluative stance” (Musolff, 2017, p. 643), subtly guiding the audience toward a preferred political or moral judgment. Similarly, “allegories” are critically significant because their ideological work is executed through the strategic selection of moral reasoning. As Sun and Xiong (2022, p. 4) argue, allegories activate different scenarios depending on the application of moral reasoning; the deliberate choice of one scenario over another within an allegorical story is a decisive act of ideological persuasion that determines the metaphor’s explanatory power. This stands in contrast to “scripts” (Schank and Abelson, 1977), which are critiqued for their apparent neutrality. While scripts denote predetermined sequences of action within frames and may appear devoid of moral judgment, a critical analysis would contend that this very claim to neutrality can be a potent ideological maneuver, masking the normative assumptions embedded within the routine. The critical framework of this study deploys the integrated concepts of “frames,” “allegories,” “scenarios,” and “scripts” as analytical tools to deconstruct the ideological functions of metaphors. Applied to the six types of conceptual metaphors identified in Chinese-English press conference interpreting, this critical lens systematically analyzes how these metaphors are mobilized to construct and disseminate specific ideologies, advance political intentions, and engage in the discursive construction of China’s national image.

#### 4.2.1 Human metaphor and the human image

“Human” metaphors are the most frequently employed conceptual metaphors in the interpreting corpus. Their cognitive mechanism is also the most complicated. This section categorizes the source domain of human metaphors into three subdomains: “human body,” “social being,” and “human psychology and cognition.” These three subdomains form the basis for analyzing metaphorical expressions within the broader cognitive domain of “human.” First, human body metaphors appear 29 times, while social being metaphors occur 159 times. These two source domains represent the most salient human metaphors. Second, the source domains related to human psychology and cognition indirectly reflect human metaphors. These semantic domains include the E (Emotion, 16 occurrences), Q (Language and Communication, 50 occurrences), and X (Psychological Actions, States, and Processes, 47 occurrences) domains. In addition, more examples demonstrating human metaphors are found in other semantic domains, such as the word “neighbor” (22 occurrences) in the H (Architecture, Houses, and Buildings) domain, which implies that China is a member of the neighborhood. This expression contains the conceptual metaphor CHINA IS A SOCIAL BEING.

Human body metaphors are extensively present in the concordance lines of “China.” For example:

1. China is a next-door neighbor with a *lips-and-teeth* relationship with the peninsula. (Excerpted from Wang Yi’s press conference in 2017)

The word “lips-and-teeth” highlights the mouth of a human. This metaphor is grounded in the cognitive domain of “human body,” with the conceptual metaphor CHINA IS A HUMAN. However, human body metaphors constitute a relatively small proportion of all the human metaphors in the interpreting corpus, with “shoulder” being the most frequent, though it appears only 7 times. Human metaphors in the interpreting corpus concentrate more on human cognition, psychology, and social actions.

Cognitive frames are hierarchical, and multileveled base-profile relationships exist within a domain matrix or frame system (Tan, 2023, p. 756). Profiling is not limited to a single cognitive domain or frame. It can also happen to a larger base, such as a domain matrix or frame system, where the profile corresponds to a subdomain or subframe (Tan, 2023, p. 756). In this profiling case, one cognitive base can act as the profile of a higher-level base. In Example (1), the word “neighbor” belongs to the H1 (Areas around or near houses) domain and conveys the conceptual metaphor CHINA IS A SOCIAL BEING. This inference is based on a two-level profiling process: “neighbor” is a profile of the cognitive base “community,” which, in turn, is a profile of the larger cognitive base “society.” “Neighbor” then suggests that China exists in a society.

The term “social being” typically refers to individuals able to interact and communicate with other human beings in a society. Most clearly, the conceptual metaphor CHINA IS A SOCIAL BEING can be inferred from the frequently occurring linguistic expressions found in the S (Social Actions) domain, which appears 159 times. For example:

1. China *supports* all efforts that are conducive to the resolution of the Korean nuclear issue through dialog and negotiation. (Excerpted from Li Keqiang’s press conference in 2018)

**Figure 7:**
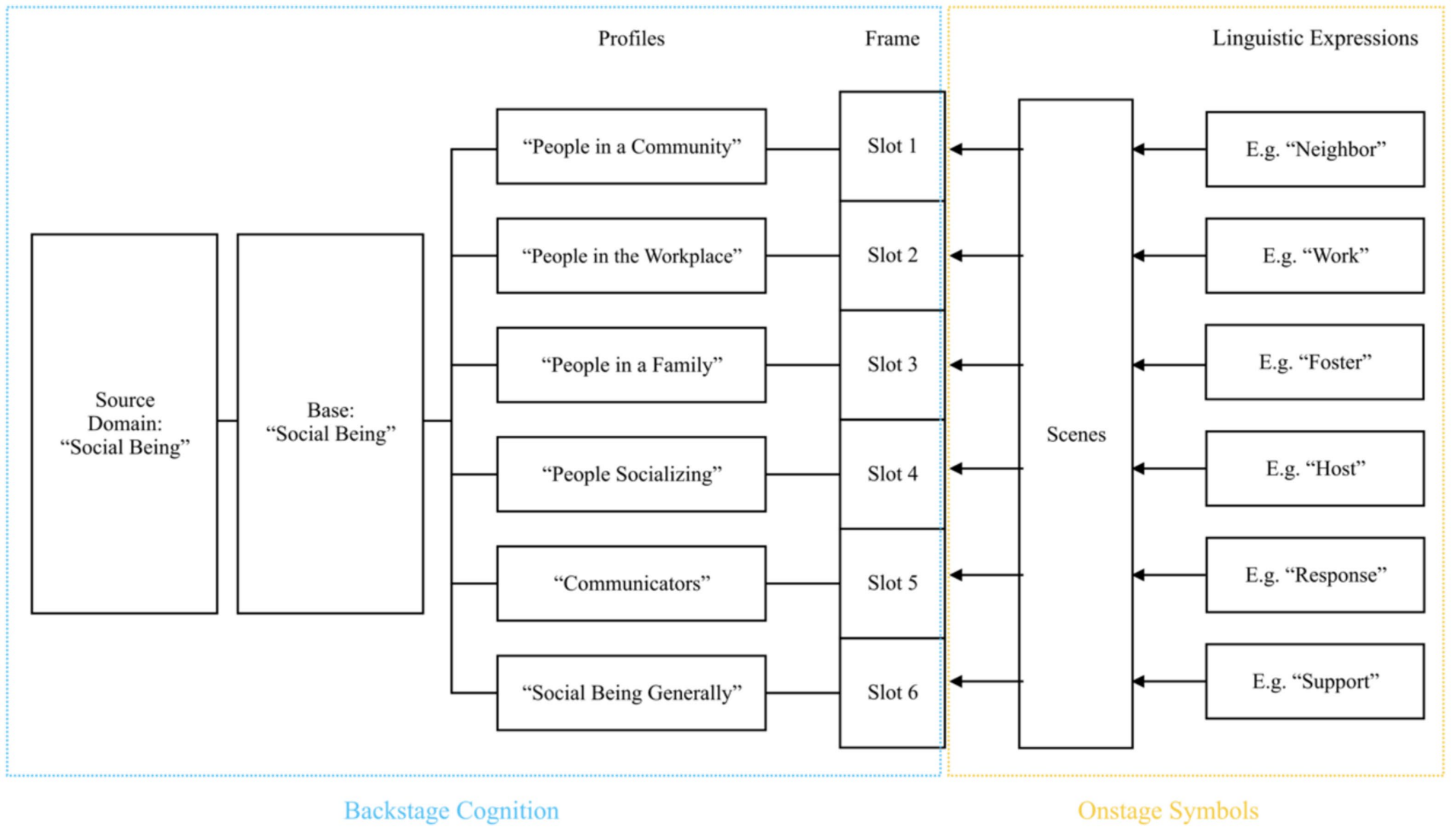
Cognitive mechanism of “Social Being” metaphors.

The word “support,” which appears 34 times in the corpus, belongs to the S8 + (Helping) domain. This social action involves a supporter and a beneficiary, indicating that China is a human in society. The social being metaphors are the most widely used human metaphors, and their linguistic expressions also span several other semantic domains, as shown in Figure 7.

Human metaphors are not necessarily tied to physical body parts or social actions. They can also be inferred from expressions in the E (Emotion), Q (Language and Communication), and X (Psychological Actions, States, and Processes) domains. These semantic domains relate to human psychological and cognitive processes, representing more abstract human qualities. For example:

1. China will adopt a more *visionary* foreign policy (Excerpted from Wang Yi’s press conference in 2018)

“Visionary” appears once in the corpus and falls within the X9.1 + (Able/Intelligent) domain, representing the profile of “human mental qualities.”

Human metaphors can construct the “nation as person frame” (Entman, 1993; Sun and Xiong, 2022, p. 7), which anthropomorphizes the state by representing national actions and diplomatic affairs through human bodily characteristics and interpersonal relations. Within this frame, state behavior is characterized by human traits, including physical, emotional, and cognitive features. In the “scenario” construction process, the interpreting discourse employs human metaphors to concretize abstract national actions via embodied experience, linking them to the scenarios of everyday interpersonal interactions familiar to the audience. In the “script” of human metaphor, the audience can anticipate the nation exhibiting human-like qualities such as socialization, communication, cooperation, and mutual assistance. For Western politics, Lakoff’s (1996/2002) Theory of Moral Reasoning (TMR) presupposes that conservatives and liberals unconsciously build their worldviews around different conceptual metaphors that describe the state as a family. Similarly, in the “allegory” of human metaphor, the relationships between nations are framed to caution that states should not be seen as cold diplomatic machines but as entities capable of conveying emotions and of interacting under the guidance of the ethics and values of human society.

Human metaphors in the interpreting discourse serve two key purposes. First, they offer vivid and engaging expressions that quickly capture the audience’s attention (Wu et al., 2020, p. 86). Diplomatic interpreting often addresses formal topics or abstract concepts, while human metaphors make the spokesperson more accessible. They resonate with the audience’s everyday embodied experience, thereby enhancing interest and reducing tension. Second, it plays a crucial role in shaping a positive image of China. Instead of being perceived as a distant and impersonal superpower, China is portrayed as a living, breathing entity with human feelings and emotions. Furthermore, the social being metaphor positions China within the international community. In interpreting discourse, China is frequently portrayed as a “helper,” “coworker,” or “supporter,” which underscores the country’s constructive role in the international community.

This preference for positively valued interpersonal roles also reflects a broader strategy of discursive naturalization in translation and interpreting. As metaphor use is adjusted for cross-cultural communication, a planned cross-lingual mass communication act occurs (Forceville, 2020), where discursive and cognitive functions of ST and TT are prioritized (Song and Zhang, 2023, p. 8). In this sense, expressions that may evoke ideological distance or outgroup perception tend to be avoided, while those aligned with universally shared social norms are foregrounded. The prominence of “supportive” and “cooperative” roles not only enhances accessibility but also contributes to a “non-distorted Self” by minimizing potentially alien or confrontational representations (Van Dijk, 2006, p. 118; Song and Zhang, 2023, p. 9).

#### 4.2.2 Journey metaphor and the traveler image

Another salient conceptual metaphor identified in the interpreting corpus is the journey metaphor, with the majority of related metaphorical expressions situated within the M (Moving and Location) domain. This metaphor occurs 251 times in total. For example:

1. The (China-Japan) relationship will *steer* clear of obstacles and interference and enjoy a stable and bright outlook. (Excerpted from Wang Yi’s press conference in 2019)

The word “steer” appears once in the corpus and belongs to the M2 (Putting, pulling, pushing, transporting) domain. The profiling process (Tan, 2023) of the “steering action” highlights the cognitive base of the “driver,” which, in turn, serves as a profile of the larger cognitive base of the “journey.” The implied conceptual metaphor is CHINA-JAPAN RELATIONSHIP IS ON A JOURNEY.

**Figure 8:**
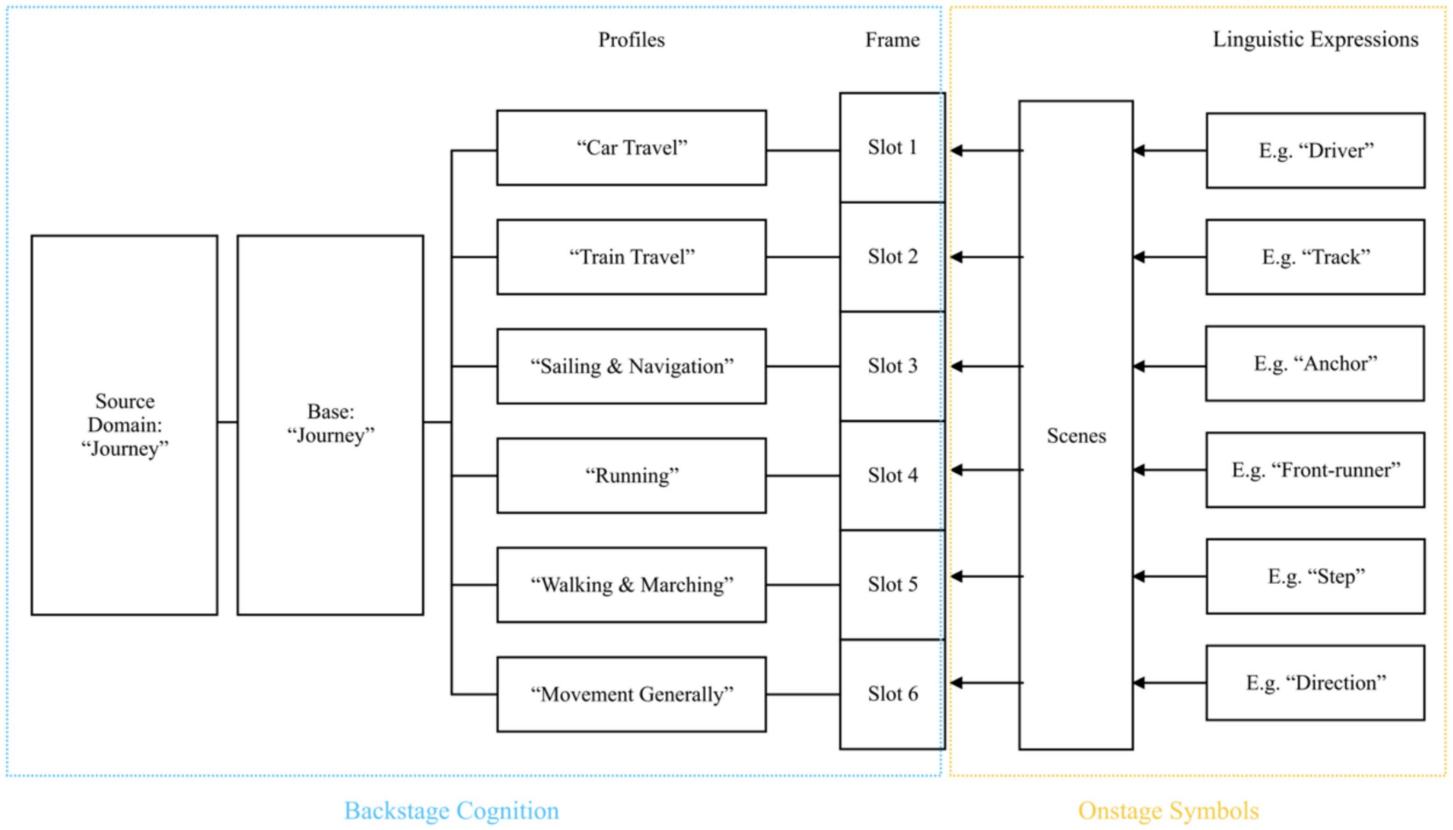
Cognitive mechanism of “Journey” metaphors.

“Journey” is an overarching cognitive base encompassing various cognitive profiles, such as driving, sailing, running, marching, climbing, flying, and even space travel. Based on the Profiling Theory (Tan, 2023), the cognitive mechanism of journey metaphors is illustrated in Figure 8.

The journey metaphor constructs the “struggle as journey frame” (Entman, 1993), where the “scenario” depicts a collective journey of China and the international community striving together. In this context, the “script” refers to a predetermined sequence of events during such a journey, such as guidance along the way, the obstacles throughout the path, and the arrival at the ultimate destination. The repetition and predictability of the script lend a clear structure to the journey metaphor, allowing the audience to understand China’s leadership role, steadfast determination throughout the journey, and clear goals in international affairs. Such structuring is not purely cognitive but also shaped by considerations of audience reception in cross-cultural communication. In this regard, the journey metaphor, as also observed in other translated Chinese political discourse, follows a norm of assimilation aimed at eliminating repetitive rhetorical terms (Deng and Zeng, 2020) and adapting to the TL readers’ rhetorical psychology (Gong, 2012). This process can be understood as a form of discursive naturalization, whereby metaphor choices are adjusted to enhance acceptability while minimizing the salience of difference.

Moreover, the journey metaphor conveys an implicit moral lesson through “allegory,” which calls on China and the international community to face challenges together and advance hand in hand to reach a bright future. Journey metaphors are common in political discourse, for they depict political life as a collective journey guided by the leaders toward a common destination (Wu et al., 2020, p. 86). Press conference interpreting employs this metaphor by characterizing China and the international community’s endeavor as a journey to serve two significant purposes: First, it highlights the challenges along the path of struggle, emphasizing China’s crucial role in providing leadership to help other countries and the international community navigate these challenges to achieve stability and success. Second, the journey metaphor emphasizes the common objectives and promising future shared between China and the global community. Every journey has a destination. Such a belief provides a unifying focus for China and other nations while instilling confidence and motivation in the world’s journey of cooperation.

#### 4.2.3 Building metaphor and the builder image

Unlike the multifarious metaphor-bearer words of the journey metaphor, “building” metaphors represent a monotonous metaphor type with a limited range of metaphor-bearer words. The only two words that carry building metaphors in the interpreting corpus are “build/building” and “pillar,” with frequencies of 43 and 2. For example:

1. China and Russia standing together will remain a *pillar* of world peace and stability. (Excerpted from Wang Yi’s press conference in 2021)

The word “pillar” belongs to H2 (Parts of buildings), and by profiling the essential part that supports the entire structure, it activates the source domain of “building.” The conceptual metaphors are CHINA IS A PILLAR and WORLD PEACE AND STABILITY IS A BUILDING.

The building metaphor is widely shared in both Chinese and Western contexts. In British political discourse, building metaphors are motivated by the conceptual metaphor SOCIETY IS A BUILDING and invariably convey a positive evaluation as a valued outcome requires social cooperation between the British government and the people (Charteris-Black, 2004, p. 71). Social goals are depicted as requiring patience, effort, and potential sacrifices without immediate outcomes, and this emphasis on long-term progress is reflected in building metaphors, which consistently convey a positive evaluation of subsequent developments (Charteris-Black, 2004, p. 71). In Donald Trump’s Gettysburg address, the building metaphor is deployed to map the image of a leading engineer (D1, the source domain) onto himself (D2, the target domain), thereby emphasizing his leadership role (Wu et al., 2020, p. 87). Furthermore, the construction of the building requires the collective effort of coworkers, thus evoking positive emotions among the audience (Wu et al., 2020, p. 87). In addition, the building metaphor includes an “up-down” orientational metaphor (Lakoff and Johnson, 1980, p. 14), where Trump described America under Obama’s administration as being “down” and promised that under his leadership, the nation would rise “up” to greatness again, signaling his intention to restore America to its peak (Wu et al., 2020, p. 87).

The building metaphor constructs the “cause as building frame,” in which the “scenario” portrays China working with other nations to achieve a shared goal. Within this metaphorical frame, the “script” includes laying the foundation, pouring cement, adding bricks, rising to greater heights, and gaining a broad perspective. The building “allegory” reflects a narrative of laying a solid foundation, constructing steadfastly, motivating fellow builders to work diligently, constantly striving for new heights, and never giving up until the building is complete. In press conference interpreting, China is depicted as a diligent builder working with other nations. It is also portrayed as a crucial pillar in the international system, which highlights its pivotal role in global affairs.

#### 4.2.4 Ecology metaphor and the guardian image

This research categorizes the source domains of ecology metaphors into three types: plants, animals, and the natural environment. The metaphorical expressions primarily fall within the L (Life and Living Things) and W (World and Environment) semantic domains, with frequencies of 8 and 4, respectively. This indicates that ecology metaphors are relatively rare in the interpreting corpus. All the metaphor-bearer words of plant and animal metaphors are in the L (Life and Living Things) semantic domain. For example:

1. After decades of diligent nurturing, the *sapling* of China-Africa cooperation has grown into a towering *tree* that no force can topple. (Excerpted from Wang Yi’s press conference in 2019)

The words “sapling” and “tree” each occur once in the corpus, and both belong to the L3 (Plants) domain. They profile two growing stages of the cognitive base of “plants,” containing the conceptual metaphor CHINA-AFRICA COOPERATION IS A GROWING SAPLING.

In addition to plant and animal metaphors, the context of “China” also covers natural environment metaphors. For example:

1. And that will be the warm *breeze* that China brings to the world. (Excerpted from Li Keqiang’s press conference in 2016)

The word “breeze” occurs once in the corpus, which belongs to the W4 (Weather) domain. These terms profile various weather conditions within the cognitive base of “natural environment,” conveying the conceptual metaphor CHINA IS PART OF THE ECOLOGICAL ENVIRONMENT.

The ecology metaphor constructs the “international environment as ecosystem frame” (Entman, 1993) and depicts a “scenario” of harmonious coexistence between humanity and nature. In this context, the “script” refers to nurturing, cultivating, and protecting the international environment, suggesting that international relations, like natural ecosystems, require collaborative efforts from all sides. The ecological “allegory” indicates that disrupting these “ecological” relationships would lead to an imbalance in the global system, underscoring the indispensability of international cooperation while implying China’s critical role in maintaining the “ecological” balance.

This ecological framing resonates strongly with the Chinese philosophical notion of *He*, which advocates “harmony in diversity” (Li, 2008; Song and Zhang, 2023, p. 12) and emphasizes balanced coexistence rather than confrontation. In the context of international communication, such metaphorical choices reflect a deeper ideological orientation that prioritizes cooperation, stability, and mutual development. Metaphor shifts in institutional translation are often guided by the “self-serving principle” and the mentality of *He*, aiming to “avoid sharpening conflicts” (Song and Zhang, 2023, p. 13) and promote a harmonious global order. In this sense, ecology metaphors not only construct an image of China as a “guardian” but also embed a culturally specific yet globally adaptable ideological stance centered on coexistence and interdependence.

At the same time, the relative scarcity yet strategic use of ecology metaphors can also be explained by considerations of discourse power and interpretability. As noted by Song & Zhang (2023, p. 10), “shared knowledge within a group facilitates mutual understanding among its members” (citing Van Dijk, 2006), but culturally embedded metaphors may not be readily accessible to international audiences when background knowledge is limited. In such cases, metaphorical expressions are either adapted or selectively deployed to “avoid invoking an exotic source domain” (Song and Zhang, 2023, p. 2) or to “eliminate undesired associations” (Song and Zhang, 2023, p. 9). Within this context, the ecological frame, especially when expressed through broadly intelligible imagery such as “tree” or “breeze,” allows China to convey its ideological emphasis on harmony and sustainability without relying on culturally opaque symbols. This reflects an effort to balance cultural specificity with global intelligibility, in line with the communicative goals of international discourse.

Of ecology metaphors, animal imagery metaphors have been found by Ye and Chen (2020) to frequently appear in the poetry of Xu Zhimo, the renowned Chinese poet. The formation of this metaphorical characteristic is rooted in the poet’s dual ecological views of traditional “anthropocentrism” and modern “ecocentrism,” which not only involve the scrutiny of animals as “others” based on human subjectivity but also imply a return to ecological ethics that transcends a singular human-centered viewpoint (Ye and Chen, 2020, p. 78). Moreover, Tenorio (2009, p. 116) argues that entities such as nations and societies are often described as organisms, particularly plants. Drawing on basic biological knowledge, one can infer certain entities and characteristics crucial to the nations’ or societies’ development and continued existence (Tenorio, 2009, p. 116). Like human metaphors, animal and plant metaphors are closely related to people’s everyday embodied experience and are so vivid and lively that they can effectively captivate audiences’ attention. China’s cooperative endeavors are frequently compared to animals or plants, suggesting their need for careful nurturing or cultivation, thereby subtly conveying a call for improving international cooperation through the concepts of ecological protection. Similarly, natural environment metaphors also utilize environmentalism and ecocentrism to convey the notion of safeguarding the global environment in the same way as protecting natural ecosystems. As China is depicted as a constituent and guardian of such an ecological environment, the cooperative message conveyed to international audiences can be vivid and compelling.

#### 4.2.5 War metaphor and the fighter image

War metaphors are not expected to dominate in diplomatic settings. However, they still appear in the interpreting corpus, primarily within the G (Government and Politics) semantic domain, with 7 occurrences. For example:

1. This year, China plans to win its *battle* against poverty. (Excerpted from Li Keqiang’s press conference in 2020)

The term “battle” appears 5 times in the corpus and belongs to the X8 + (Trying hard) domain. The cognitive frame of “warfare” is activated by profiling the concept of “battle,” conveying the conceptual metaphor CHINA’S POVERTY ALLEVIATION IS A WAR.

The war metaphor invokes corresponding frames to depict the sufferings the Chinese people endured decades ago through vivid imagery, in order to underscore the preciousness of hard-won peace and development, while calling for collective effort toward a brighter future (Song and Zhang, 2023, pp. 7–8). War metaphors personify abstract concepts like the pandemic and poverty as enemies, constructing a “war frame” where the nations are united in their struggle against these foes. This metaphor constructs a “scenario” of conflict and unity, with a “script” of struggles and the eventual victory. In this script, the nations must confront challenges, display strength, solidarity, and courage to ultimately triumph over threats, self-defense, and sacrifice. The war “allegory” of a desire for peace further underscores the moral legitimacy and justice of a nation’s fight against these external threats. Additionally, the war metaphor conveys a moral warning through the allegory of resistance that only through cooperation and unwavering determination can these wars without gunfire be won in international affairs.

War metaphors are common in political discourse. In the speakers’ depictions, the enemy poses various threats to the allies, necessitating united defense efforts that inevitably involve bloodshed or sacrifice (Wu et al., 2020, p. 85). Such metaphors in political discourse are primarily used to boost morale and appeal for unity in resistance rather than to make more enemies. Therefore, these metaphors must be used judiciously in diplomatic settings. In the interpreting corpus, the metaphorical enemy is limited to abstract negative concepts such as “pandemics,” “poverty,” “slanders,” or “political viruses.” For example, the interpreting discourse uses “the battle against COVID-19” by framing other dialog partners as allies, signaling a willingness to cooperate with other countries and simultaneously indicating China’s resolve and courage to win this battle throughout its struggle. The war metaphor portrays China’s national image as a fearless fighter.

This cautious use of war metaphors reflects a broader tendency toward mitigation in institutional discourse. As observed in political translation, metaphors that may evoke violence or hostility are often softened or recontextualized to avoid undesirable interpretations (Li and Pan, 2020). For instance, more sanguinary imagery may be replaced by less confrontational expressions in order to maintain a balanced tone. Such adjustments align with the self-serving principle underlying the Ideological Square Model, whereby negative implications are downplayed to preserve a favorable national image (Van Dijk, 1998). In this way, war metaphors in the interpreting corpus are carefully constrained to emphasize determination and resilience without triggering perceptions of aggression.

The necessity of such mitigation is further evidenced by cases where metaphorical expressions have led to misinterpretation in international contexts. For instance, culturally specific metaphors such as “red gene” have been reinterpreted in foreign media as signals of militaristic or expansionist intent, thereby amplifying negative other-representation (Gabbatt, 2020). This demonstrates that metaphorical expressions, when detached from their original cognitive and cultural environment, may generate unintended ideological effects. Consequently, institutional interpreters tend to avoid or attenuate metaphorical elements that could be perceived as aggressive or confrontational, ensuring that the projected image remains aligned with China’s stated commitment to peaceful development.

#### 4.2.6 Entertainment metaphor and the performer image

This section categorizes entertainment metaphors in the interpreting corpus into two types: “sports competition” metaphors and “theatrical performance” metaphors. The metaphor-bearer words of both metaphors belong to the K (Entertainment and Sports) semantic domain, with a total frequency of 45. For example:

1. China will not *play* the bully. (Excerpted from Wang Yi’s press conference in 2016)

The term “play” in Example appears 14 times, belonging to the K4 (Drama, the theatre, and show business) domain. It frames China as a character in a theatrical performance, with the profile of “acting” within the cognitive base of “theatrical performance.” The corresponding conceptual metaphor is CHINA IS A PERFORMER ON STAGE.

Donald Trump’s address at Gettysburg mapped the competitive spirit of sports from the athletic domain to the political domain (Wu et al., 2020, p. 84). The image of the United States was described as a runner falling behind others in a race. In this way, Trump wanted to ignite public morale and encourage citizens to actively participate in the 100-day plan in the same way as players compete in high spirits (Wu et al., 2020, p. 84). In diplomatic settings, the key role of sports competition metaphors is to inspire the determination of nations in their efforts toward development. Like theatrical performance metaphors, they vividly resonate with people’s everyday experiences of watching sports or plays, making it easier for audiences to understand China’s critical role in international affairs.

The entertainment metaphor likens the struggles between nations to sports competitions or theatrical performances, constructing a “frame” of entertainment activities. In the “scenario” construction process, entertainment metaphors compare the international environment to an arena or stage, with cooperative efforts between nations portrayed as athletes striving for victory on the field or performers seeking to captivate audiences on the stage. The “script” of entertainment metaphor refers to the processes of competition or performance that are familiar to the audiences, where it is anticipated that athletes and performers will demonstrate a spirit of perseverance and excellence, thereby motivating China and other countries to exhibit fighting spirit and actively engage in healthy global competitions. The competition “allegory” reveals China’s responsibility on the “field,” emphasizing its aim to achieve victory and to promote a spirit of fair competition that fosters overall development, thereby highlighting China’s responsibilities and competitiveness in international affairs. Depicting China as a determined athlete striving for victory proactively foregrounds positive in-group attributes such as perseverance, competitiveness, and responsibility—what Song & Zhang (2023, p. 13) call a “non-distorted and well-portrayed Self.” Conversely, potential negative associations (e.g., aggressive rivalry) are suppressed or reframed as “fair competition” and “joint development,” aligning with the “self-serving principle” (Li and Pan, 2020) that guides institutional discourse. Similarly, the performance allegory illustrates China’s role on the “stage,” underscoring its commitment to work alongside other performers to present the most spectacular collaborative performance to the audience. This indicates that China intends to play a key role in the “performance” by showcasing its strengths while promoting joint development.

### 4.3 Metaphor interpreting strategies and factors influencing metaphor choices

Table 7 presents the standardized frequencies of the three strategies and their frequencies within specific source domains.

**Table 7:** Standardized frequencies of metaphor interpreting strategies.

Source domains	Metaphor creations	Metaphor retentions	Metaphor transformations
A (General and Abstract Terms)	1.12	0.39	0
B (Human Body)	5.62	3.71	0
C (Arts and Crafts)	0.56	0.2	0.75
E (Emotion)	0.56	2.93	0
F (Food and Agriculture)	1.69	0.59	0
G (Government and Politics)	0.56	0.98	0.75
H (Architecture, Houses and Buildings)	5.62	10.55	11.19
I (Industry and Money)	3.37	0	14.18
K (Entertainment and Sports)	11.8	3.52	4.48
L (Life and Living Things)	0.56	1.37	0
M (Moving and Location)	38.76	32.42	11.94
N (Numbers and Measurement)	6.74	2.54	0.75
O (Substances, Materials and Objects)	2.25	1.56	1.49
Q (Language and Communication)	2.25	5.86	11.94
S (Social Actions)	16.85	21.09	15.67
T (Time)	0.56	3.12	2.99
W (World and Environment)	0	0.78	0
X (Psychological Actions, States and Processes)	1.12	8.4	1.49

In general, the proportions of Metaphor Creation (21.67%), Retention (62.11%), and Transformation (16.22%) yield an approximate ratio of “1.3:3.8:1” in strategy use. The results show that Metaphor Retention is the most commonly used strategy in press conference interpreting. The upcoming sections analyze the metaphors frequently created, retained, or transformed. The ultimate goal is to reveal the active role of press conference interpreting in discursively constructing a favorable image of China. The other goal is to unpack the factors influencing metaphor choices and explore the interplay between individual or social resources and metaphorical expressions in press conferences.

**Figure 9:**
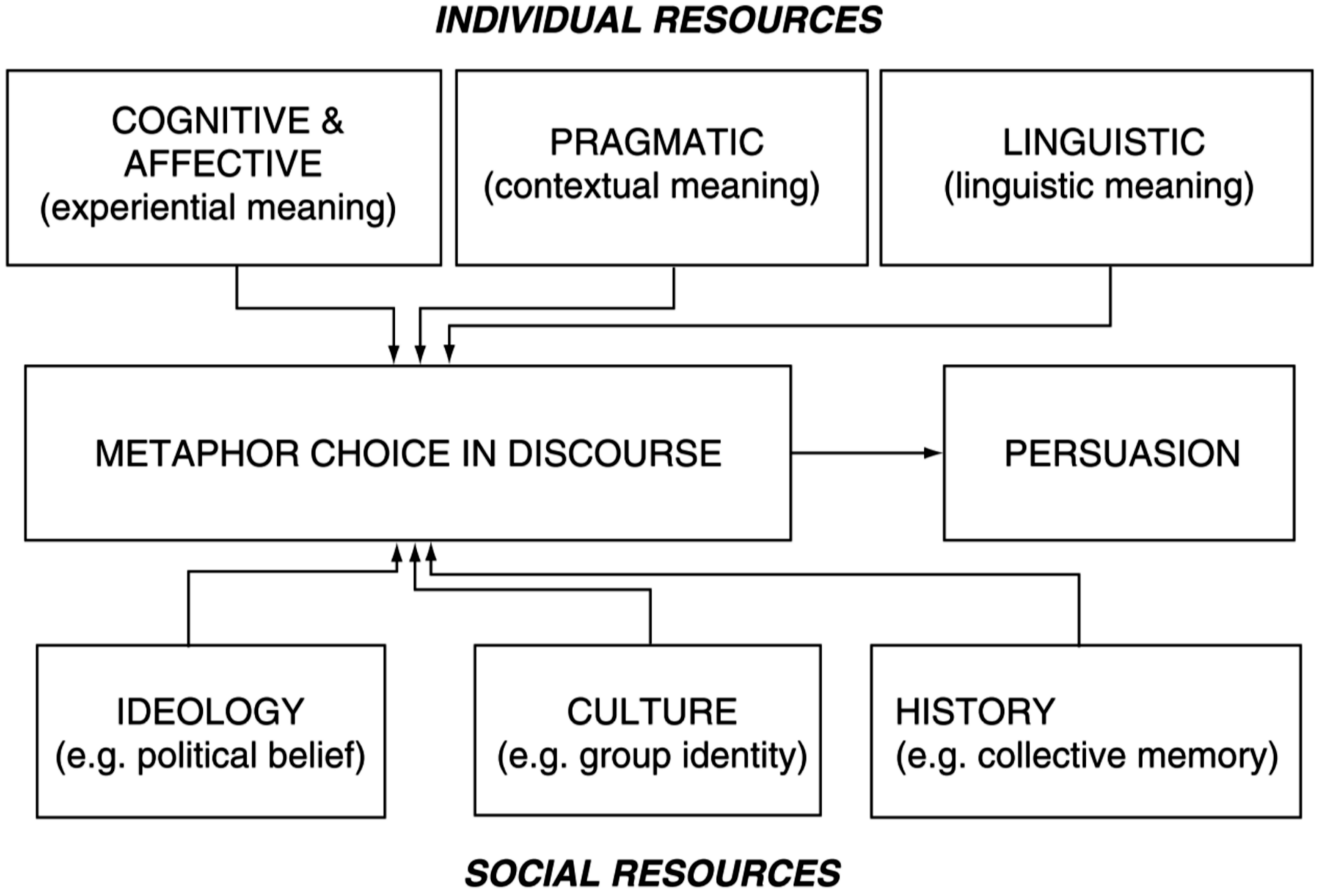
A discourse model for metaphor (Charteris-Black, 2004, p. 248).

Speakers strategically employ metaphors to achieve communicative purposes within a particular context (Charteris-Black, 2004, p. 247). The choice of metaphor can be guided by a range of considerations, including cognitive, semantic, pragmatic, ideological, cultural, and historical dimensions (Charteris-Black, 2004, p. 248). Two kinds of resources can influence metaphor choices, as shown in Figure 9.

The individual resources are categorized into three main components: the speaker’s cognitive, emotional, and embodied experiences of the world; their awareness of what is likely to be effective in specific communicative contexts; and their understanding of the linguistic system, including lexical fields and word meanings (Charteris-Black, 2004, p. 248). The social resources are subdivided into two components: the ideological outlook, typically political and religious beliefs; and the broader historical and cultural knowledge (Charteris-Black, 2004, p. 248). A combination of metaphor explanation from the previous section and Charteris-Black’s (2004) discourse model helps reveal the factors influencing metaphor choices in press conference interpreting. Moreover, translation and interpreting norms (Toury, 2012) should also be highlighted as crucial factors influencing metaphor choices, even though they may be considered part of the broader category of social resources. The following sections explore the factors influencing metaphor choices from diverse perspectives, including cognitive linguistics, social contexts, ideologies, cultures, histories, and interpreting norms.

#### 4.3.1 The creation of journey metaphors

Metaphor Creation is similar to “metaphorization” introduced by Sheng (2021) for metaphor interpreting strategies. It refers to cases where a non-metaphorical expression in the source text is translated into a metaphorical expression in the target text or where a conceptual metaphor is added in the target text without a corresponding expression in the source text (Sheng, 2021, p. 69). The majority of the created metaphors by interpreting discourse are found in the M (Moving and Location) semantic domain. This indicates that China’s development or collaborative efforts with other countries are frequently conceptualized as a journey. The most commonly created metaphorical expressions by interpreting discourse are “ahead” (11 times), “step” (8 times), and “course” (8 times). For example:

1. ST: 今天的中国正在加快*实现*“两个一百年”的重要目标。(Excerpted from Wang Yi’s press conference in 2014)

TT: Today’s China is *marching* ever faster toward what we call the “two centenary goals.”

1. ST: 无论国际风云如何变幻，中国都会坚定不移地扩大开放。(Excerpted from Li Keqiang’s press conference in 2022)

TT: No matter how the international environment may change, China will keep to the *course* of wider openness.

**Figure 10:**
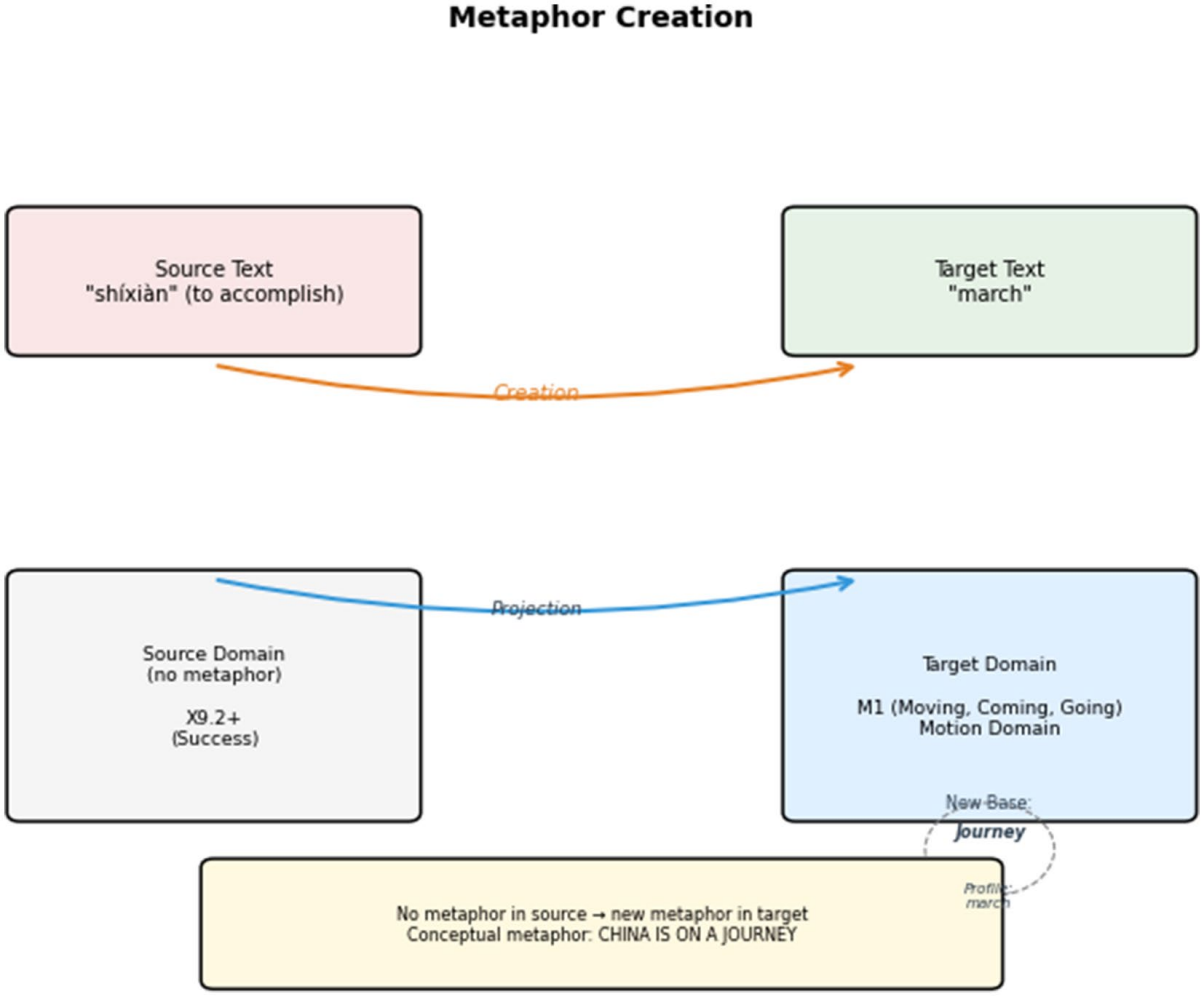
Cognitive mechanism of metaphor creation.

The original Chinese texts contain no journey metaphor, while the interpreting discourse has used the metaphor-bearer words “marching” and “course” to create journey metaphors. In Example (1), the source text uses “实现 (to accomplish),” which belongs to the X9.2 + (Success) domain (see USAS tagset, Wmatrix5) and carries no conceptual metaphor. The interpreting discourse uses “march” in the M1 (Moving, Coming, and Going) domain. The created conceptual metaphor is CHINA IS ON A JOURNEY. The cognitive schemata and pathways that underpin Metaphor Creation are shown in Figure 10.

The “metaphorization” (Sheng, 2021) of the journey concept in the target language might imply a positive evaluation of the related policy (Charteris-Black, 2004, p. 95). When no metaphor is present in the source text, the addition of a journey metaphor can enhance the coherence of the target text while assisting in constructing a narrative frame (Musolff, 2006, p. 36). The knowledge associations activated by the metaphorical narrative are essential for gaining the target audience’s agreement and achieving the persuasive function of the interpreted discourse (Sheng, 2021, p. 69). This suggests that China’s image has been portrayed as a traveler or navigator on a journey. This metaphor choice highlights China’s confidence and significant leadership role in its development and international cooperation efforts while emphasizing its steadfast determination to advance toward shared global goals.

The first factor influencing the creation of journey metaphors is the shared embodied cognition of journeys. Based on the Embodied Philosophy (Lakoff and Johnson, 1999), the journey metaphor shares many commonalities between Chinese and English. The significance of this metaphor in people’s lives stems from its entailments, which are shaped by shared cultural knowledge of journeys (Lakoff and Johnson, 1999, p. 62). For example, journeys may involve challenges that one is expected to foresee and prepare for. A PURPOSEFUL LIFE IS A JOURNEY is another more frequently used conceptual metaphor. Based on the foundational metaphors PURPOSES ARE DESTINATIONS and ACTION IS MOTION, a shared cultural understanding combines the belief that individuals should have a life purpose and the conceptualization of a long journey as movement across multiple destinations (Lakoff and Johnson, 1999, p. 62). By incorporating shared embodied cognition, China’s image as a determined traveler on a journey is reinforced.

Chinese cultural traditions, such as the value placed upon the concept of “Tao” (the path) and the notion of enduring hardship in pursuit of long-term goals, contribute to the widespread use of journey-related imagery. This cultural foundation reinforces the idea of a nation on an ongoing journey, constantly progressing toward a better future. Historically, China’s experience of overcoming adversity, particularly in the context of its rapid modernization and development in the 20th and 21st centuries, further strengthens the journey metaphor. The country’s historical narrative of overcoming economic hardship serves as a backdrop to the journey metaphor, which frames China’s development as an enduring and transformative journey.

Cognitively and discursively, these salient additions also constitute cases of positive self-representation (Van Dijk, 1984) on the part of the government-affiliated interpreters in reconstructing a firmer, more confident, and decisive image of China in its development push, rather than one that is weak, hesitant, or equivocal (Gu, 2022, p. 75). This tendency can be further understood in light of metaphor shifts as a process of discursive naturalization in cross-cultural communication. As Forceville (2020) notes, translating political discourse into another language constitutes a planned cross-lingual and cross-cultural mass communication act, in which audience reception becomes a primary concern. In this process, metaphorical expressions that align with shared social cognition are more likely to be retained or foregrounded, whereas those that may signal ideological distance are avoided. Such selective representation contributes to what van Dijk (2006, p. 118) describes as the effort to eliminate inconsistencies between ideological beliefs and perceived realities, thereby ensuring a coherent and acceptable Self in international discourse.

#### 4.3.2 The creation and transformation of human metaphors

The frequent use of Metaphor Creation is also evident in the S (Social Actions) semantic domain, which includes various human metaphors, particularly the social being metaphors. This suggests that China’s image is often conceptualized as a human living in society. The three largest human metaphors created by the interpreting discourse are “help” (10 times), “host” (8 times), and “foster” (3 times). For example:

1. ST: 我们希望美方摒弃零和思维，同中方相向而行，在深化合作的进程中*形成*良性竞争，在各自发展的同时实现互利双赢。(Excerpted from Wang Yi’s press conference in 2019)

TT: We hope the US will reject its zero-sum approach, pursue shared goals with China, *foster* healthy competition while deepening our cooperation, and strive for win-win outcomes as we seek our own countries’ development.

The word “形成” has a literal translation of “form,” which carries no conceptual metaphor. The interpreting discourse uses “foster” to create the human metaphor with the source domain of “human in a family.” The competition between China and the US is then viewed as a child to foster, where the “family scenario” is framed. The family metaphor is widely used in news or social media because it evokes strong emotional resonance (Sun and Xiong, 2022, p. 5). According to Lakoff’s (1996/2002) Theory of Moral Reasoning (TMR), Western liberals’ worldview originates from the metaphorical frame of the state as a nurturing parent, in which morality is based on empathy and compassion (Burgers et al., 2016, p. 414). Within a similar family frame, the interpreting discourse portrays China and the U. S. as nurturing parents, whose healthy competition is compared to a child being fostered. This metaphor conveys China’s sense of responsibility as a parent. The creation of human metaphors indicates that press conference interpreting shapes China’s image as a socially connected human who is intricately linked with other global actors and frequently plays positive roles such as “hosting” or “fostering.” This helps reflect China’s active and caring attitude toward participating in and supporting various international affairs.

Schäffner (2004) suggests that the shift between two conceptual metaphors may occur because both can be subsumed under a broader conceptual mapping. For example, the architecture and journey metaphors are interchangeable, as both emphasize participants’ patience and perseverance, requiring sacrifices rather than expecting quick results (Charteris-Black, 2004, p. 96). Nevertheless, from a cognitive linguistic perspective, the original metaphor’s “cognitive base” (Langacker, 1987; Tan, 2023) has been changed in this case, leading to profound changes in the conceptual meaning. This study finds that the interpreting discourse frequently transforms metaphors into the S (Social Actions) domain, with “foster” being a high-frequency metaphor-bearer word (transformed seven times in the interpreting corpus). For example:

1. ST: 我们要同各国一道，共同建设新型国际关系。(Excerpted from Wang Yi’s press conference in 2018)

TT: China will work with other countries to *foster* a new type of international relations.

**Figure 11:**
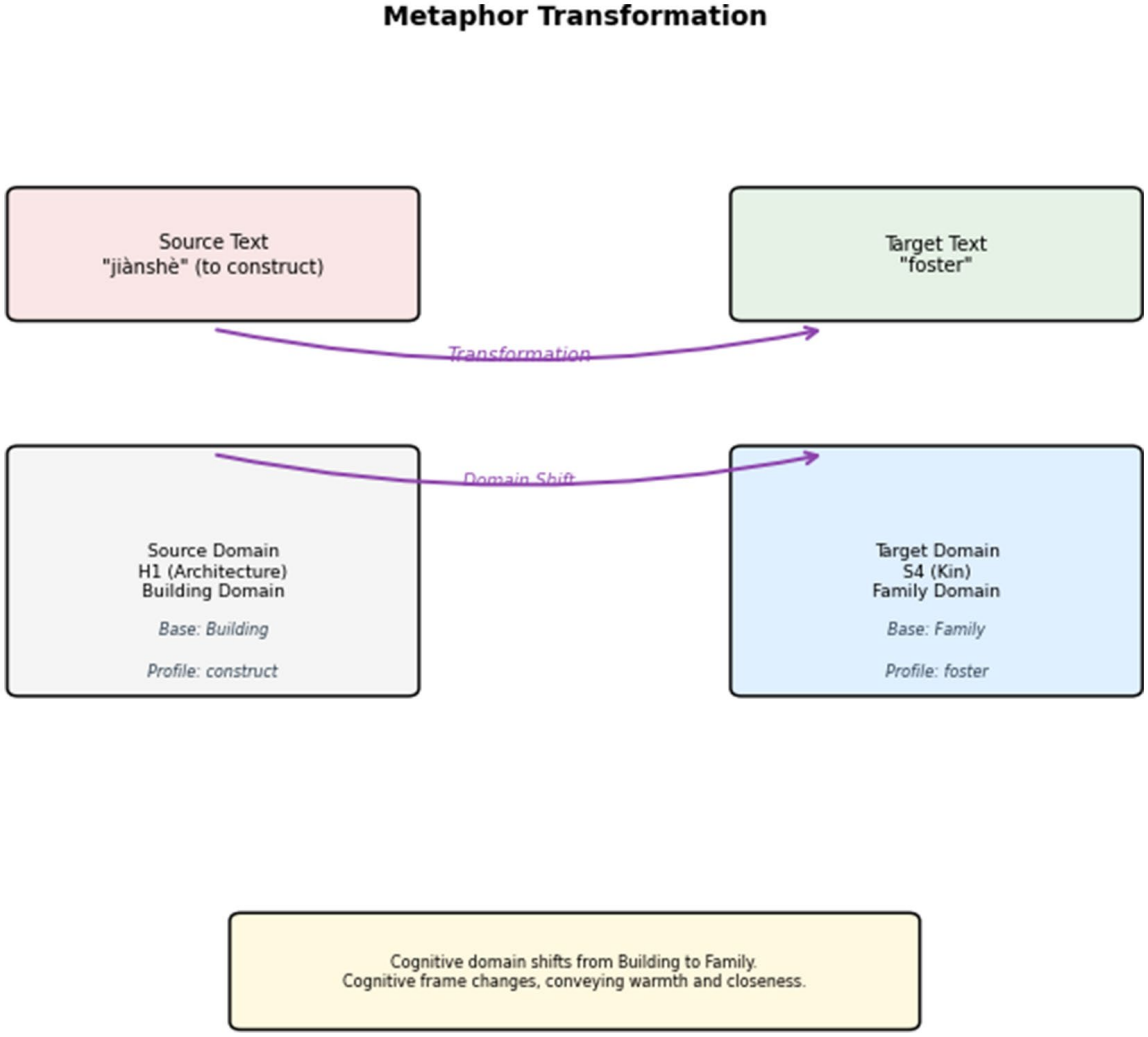
Cognitive mechanism of metaphor transformation.

The interpreting discourse translates “建设 (construct/build)” as “foster,” which marks a shift from the cognitive subdomain of the H1 (Architecture, Houses and Buildings) to the S4 (Kin) domain. This metaphor choice is based on the similarity between “building” and “fostering,” as both concepts require patience and perseverance. However, the profiling process is deliberately situated within the cognitive base of “family” instead of “construction,” which signifies a subtle conceptual difference that press conference interpreting intends to create. Metaphor Transformation depicts China’s image as a global family member who fosters closeness and warmth, reinforcing the idea that China and other countries are as intimate as family members. As “family member” is a profile of the larger cognitive base of “social being,” which again serves as a profile in the cognitive base of “human,” according to the hierarchical feature of cognitive frames (Tan, 2023, p. 756), this transformed metaphor is also classified as a human metaphor. The cognitive schemata and pathways that underpin Metaphor Transformation are shown in Figure 11.

The family metaphor is a prevalent type of metaphor in news and social media, largely due to its ability to evoke strong emotional resonance as people rely on their daily experiences of close relationships with family and friends to understand the dynamics between groups, organizations, and nations (Sun and Xiong, 2022, p. 5). When nations are metaphorically depicted as a big family, the “family relation” must be founded on equality, cooperation, and mutual benefits in international relations (Sun and Xiong, 2022, p. 7). In this context, each country diminishes its unique characteristics as an individual entity and instead finds a sense of identity and belonging as a member of the big family. This leads China, whose image as a family member is emphasized, to advocate for genuine collaboration, where nations work side by side for the collective good of the family.

The creation and transformation of human metaphors are profoundly influenced by shared embodied cognition (Lakoff and Johnson, 1999), as the spokesperson, interpreter, and audience are all real, tangible human beings existing in a large society. Embodied cognition is one of the key factors that provide the foundation for creating, conveying, and understanding human metaphors. The human body, social actions, daily experience, and mental states exhibit a high degree of universality across languages and cultures. Moreover, similar to ecology metaphors, human metaphors can also effectively disseminate moral ideals. People’s morality about human well-being is conceptualized and communicated by an extensive system of metaphoric mappings that populates the cognitive unconscious (Lakoff and Johnson, 1999, p. 290). The use of “foster” endows China with human emotions, thereby subtly and even unconsciously conveying the legitimacy and moral sensibility of building healthy competition. This created metaphor simultaneously constructs the image of China as a responsible and committed human being in the international society. Culturally, Confucian values that stress relational ethics, filial piety, and moral responsibility reinforce the construction of China as a responsible human or social being. These traditions encourage metaphors in which the nation is personified as a moral leader, a trustworthy companion, and more roles imbued with emotional resonance and ethical expectations.

#### 4.3.3 The creation of entertainment metaphors

In the interpreting discourse, a large number of “entertainment” metaphors are created, primarily utilizing the metaphorical expressions “play” and “player,” which fall within the K (Entertainment and Sports) semantic domain and have been created 16 times in the corpus. For example:

1. ST: 中欧代表着多极世界两支重要*力量*，双方关系是平等、开放的，不针对第三方，也不受制于第三方。(Excerpted from Wang Yi’s press conference in 2021)

TT: China and Europe are two important *players* in this multipolar world. The relationship is equal and open, not targeting any third party or controlled by anyone else.

In the source text, “力量” is an abstract term and contains no entertainment metaphor, whereas “player” in the target text carries the conceptual metaphor CHINA IS A PLAYER ON THE FIELD. China’s image is constructed as a key player in the international arena. Moreover, the creation of entertainment metaphors is partly due to the linguistic differences between Chinese and English. The frequent use of expressions like “play a (constructive/active) role” when interpreting phrases like “发挥重要作用” highlights a common English idiomatic structure in the interpreting discourse, which is a highly desirable choice under time constraints. This approach enables the quick conveyance of the spokesperson’s intended meaning while enriching and vividly portraying China’s national image as a dynamic and proactive actor on the global stage.

The created entertainment metaphors in interpreting, particularly those related to performance and sports, are shaped by political, cultural, and historical factors. Politically, portraying China as a “player” or “performer” on the global stage supports its strategic narrative of being an engaged and competitive participant in international affairs. This metaphorical framing helps highlight China’s soft power and confident posture, especially in diplomatic settings. Culturally, such metaphors resonate with longstanding Chinese values rooted in Confucianism, where fulfilling one’s role with propriety and grace is seen as essential to maintaining harmony and social order. The well-known Chinese saying, “One minute on the stage takes ten years of practice,” underscores not only the intense effort behind each performance but also the significance of the performance itself for both the audience and the performer. These values lend natural support to metaphors that emphasize on-stage coordination and performance. Historically, as China has evolved from a relatively peripheral actor to a great power in global governance, metaphors associated with organized games or theatrical performance reflect the country’s growing confidence and desire for recognition. This shift contributes to constructing a more competitive and credible image of China on the global stage.

This tendency can also be understood as part of a broader strategy of ideological mediation in institutional translation. Translating political discourse is not merely a linguistic operation but a process shaped by collective decision-making and guided by the “self-serving principle” (Li and Pan, 2020), whereby discourse is adjusted to serve national interests. In this process, metaphorical expressions that reduce ideological tension and enhance audience engagement are preferred. Entertainment metaphors, by drawing on culturally neutral and widely shared experiential domains, effectively lower the ideological salience of political messages, allowing them to be received as common-sense or universally relatable narratives rather than overtly political claims.

#### 4.3.4 The retention of journey and human metaphors

Sheng (2021) has addressed a similar Metaphor Retention issue through the concept of “metaphor equivalence” (p. 67). When the target text and source text share the same conceptual metaphor and metaphorical expression, the interpreter transfers the conceptual metaphor and its expression from the source text to the target text, preserving the mapping relationship and imagery presented in the source metaphor (Deignan et al., 1997). This strategy swiftly achieves “cognitive equivalence” (Katan, 1999) between the source and target metaphors, allowing the target audience to receive the metaphors through the same conceptual mappings and imagery as the source audience. This approach is considered the “optimal strategy” in metaphor interpreting and represents the ideal state of equivalence proposed by Newmark (1982). From a cognitive linguistic perspective, the two poles (i.e., the source and target domains) of the conceptual mapping (Tan, 2016, p. 97) remain unchanged, though the profiles of the cognitive base within these domains may differ.

Interpreters most frequently retain metaphors in the M (Moving and Location) and S (Social Actions) domains, with 166 and 108 retention frequencies, respectively, representing the two highest frequencies in the interpreting corpus. For example:

1. ST: 中国将坚定不移地走和平发展*道路*。(Excerpted from Li Keqiang’s press conference in 2016)

TT: China will remain committed to the *path* of peaceful development.

1. ST: 解决半岛核问题不能只有一手，需要两手并进。(Excerpted from Wang Yi’s press conference in 2017)

TT: To resolve the nuclear issue, we (China and other countries) have to walk on both *legs*.

**Figure 12:**
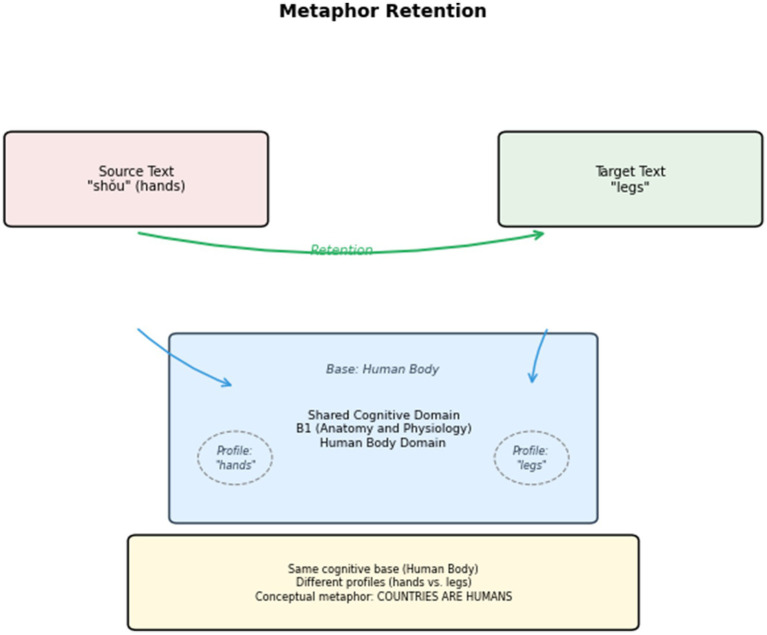
Cognitive mechanism of metaphor retention.

In Example (1), the interpreting discourse retains the journey metaphor by profiling the same concept of “path” within the cognitive base of “journey.” In Example (2), the source text uses “手 (hands),” which belongs to the B1 (Anatomy and Physiology) domain, and the interpreting discourse uses “legs” in the same domain. These two words reveal different profiles in the same underlying base, yet the cognitive domain remains the same. The conceptual metaphor remains COUNTRIES ARE HUMANS. The cognitive schemata and pathways that underpin Metaphor Retention are shown in Figure 12.

Metaphor Retention cannot necessarily prove the conscious effort made by interpreting discourse to shape a specific national image of China. However, it suggests that Chinese spokespersons prefer the journey and human metaphors and that official interpreters frequently use Metaphor Retention to convey the majority of these metaphors (approximately 66% in the semantic domain of “Moving and Location” and around 68% in “Social Actions”). This demonstrates a significant similarity between metaphorical expressions in English and Chinese within these two cognitive domains, where retaining metaphors or applying “metaphor equivalence” (Sheng, 2021, p. 67) can swiftly achieve cognitive equivalence. Metaphor Retention is a highly desirable and optimized strategy for interpreting conceptual metaphors, as it ensures both fidelity to the source message and coherence in the target language.

## 5 Conclusion

Based on the Chinese-English Press Conference Interpreting Corpus, this study employs Critical Metaphor Analysis and cognitive linguistic theories to investigate the conceptual metaphors of the term “China” in press conference interpreting. Three main findings emerge. First, six types of metaphors are identified: human, journey, building, ecology, war, and entertainment metaphors. Second, these metaphors collectively construct a multifaceted and positive image of China in the international community. Specifically, China is portrayed as a traveler on a journey of struggle, a contributor to the international community, a builder of global causes, a guardian of the global environment, a fighter in challenging endeavors, and a performer on the world stage. Third, metaphor retention remains the most common interpreting strategy. Interpreters tend to transform a range of metaphors into human metaphors, and they actively create human, journey, and entertainment metaphors to render China’s image more vivid and warm.

This study makes three contributions to critical metaphor studies in interpreting contexts. First, it demonstrates that conceptual metaphors in press conference interpreting are not merely cognitive devices for making abstract ideas more accessible. They serve as sites of ideological mediation that perform crucial ideological work. By examining the six metaphor types, the study shows how each contributes to constructing a particular narrative about China’s national image. Second, the study extends the growing body of research on institutional interpreters’ ideological mediation by showing that metaphors are particularly potent vehicles for ideological expression. Metaphors operate at a level below conscious awareness while shaping cognitive frameworks, making them especially effective in guiding audience interpretation. Third, the study adapts the CMA model to investigate the relationship between cognitive mechanisms and ideological functions. The adapted model illustrates how linguistic expressions activate cognitive frames, which in turn perform ideological work by profiling within the cognitive base. This integration of cognitive and critical perspectives offers a more comprehensive analytical framework for future research on metaphor in political discourse.

A limitation of this study is its exclusive focus on the node word “China” as the entry point for metaphor identification. While this approach enables systematic and replicable analysis, it necessarily excludes metaphorical constructions of the nation that do not directly collocate with this term. Future research could extend the analysis by examining metaphorical expressions associated with pronouns (such as “we” and “our”), metonymic references (such as “Beijing” and “the Chinese government”), and other referential strategies that contribute to the discursive construction of China’s national image. Such research would provide a more comprehensive understanding of how metaphors function in press conference interpreting to construct national images.

## Data Availability

The raw data supporting the conclusions of this article will be made available by the authors, without undue reservation.

## Footnotes

1
https://ucrel-wmatrix5.lancaster.ac.uk/wmatrix5.html
